# Targeting Treg cells with GITR activation alleviates resistance to immunotherapy in murine glioblastomas

**DOI:** 10.1038/s41467-021-22885-8

**Published:** 2021-05-11

**Authors:** Zohreh Amoozgar, Jonas Kloepper, Jun Ren, Rong En Tay, Samuel W. Kazer, Evgeny Kiner, Shanmugarajan Krishnan, Jessica M. Posada, Mitrajit Ghosh, Emilie Mamessier, Christina Wong, Gino B. Ferraro, Ana Batista, Nancy Wang, Mark Badeaux, Sylvie Roberge, Lei Xu, Peigen Huang, Alex K. Shalek, Dai Fukumura, Hye-Jung Kim, Rakesh K. Jain

**Affiliations:** 1 Edwin L. Steele Laboratories, Department of Radiation Oncology, Massachusetts General Hospital (MGH) and Harvard Medical School (HMS), Boston, MA USA; 2 Department of Cancer Immunology and Virology, Dana-Farber Cancer Institute (DFCI) and Harvard Medical School, Boston, MA USA; 3 Department of Chemistry, Institute for Medical Engineering & Science, and Koch Institute for Integrative Cancer Research, Massachusetts Institute of Technology (MIT), Cambridge, MA USA; 4 Broad Institute of MIT and Harvard University, Cambridge, MA USA; 5 Ragon Institute of MGH, MIT & Harvard, Cambridge, MA USA; 6 Program in Health Sciences and Technology, Harvard Medical School, Boston, MA USA; 7 Department of Immunology, Harvard Medical School, Boston, MA USA

**Keywords:** CNS cancer, Tumour immunology, Tumour immunology

## Abstract

Immune checkpoint blockers (ICBs) have failed in all phase III glioblastoma (GBM) trials. Here, we show that regulatory T (Treg) cells play a key role in GBM resistance to ICBs in experimental gliomas. Targeting glucocorticoid-induced TNFR-related receptor (GITR) in Treg cells using an agonistic antibody (αGITR) promotes CD4 Treg cell differentiation into CD4 effector T cells, alleviates Treg cell-mediated suppression of anti-tumor immune response, and induces potent anti-tumor effector cells in GBM. The reprogrammed GBM-infiltrating Treg cells express genes associated with a Th1 response signature, produce IFNγ, and acquire cytotoxic activity against GBM tumor cells while losing their suppressive function. αGITR and αPD1 antibodies increase survival benefit in three experimental GBM models, with a fraction of cohorts exhibiting complete tumor eradication and immune memory upon tumor re-challenge. Moreover, αGITR and αPD1 synergize with the standard of care treatment for newly-diagnosed GBM, enhancing the cure rates in these GBM models.

## Abstract (web summary)

Glioblastomas (GBM) are frequently resistant to immune checkpoint blockade therapy. Here the authors show that treatment with an agonistic anti-GITR antibody converts tumor infiltrating regulatory T cells to effector cells, overcoming resistance to PD1 blockade in preclinical models of GBM.

## Introduction

Glioblastomas (GBMs) are resistant to immune checkpoint blockers (ICBs) that have revolutionized the treatment of other solid malignancies. Although there are some reports of GBM patients responding to ICBs in phase II/III trials, ICBs have failed in all phase III randomized trials in both newly diagnosed and recurrent GBM patients to date^1–3^. The clinical benefits were observed in <10% of patients, and only when ICBs were administered in the neoadjuvant (pre-operative) setting in phase I/II trials^4,5^. This limited efficacy of ICBs is primarily due to the highly immunosuppressive tumor microenvironment (TME) of GBM^1^. ICBs such as anti-programmed cell death 1 antibody (αPD1) reinvigorate the tumoricidal activities of dysfunctional tumor-infiltrating cytotoxic CD8 T lymphocytes (CTLs)^6^. GBMs are in general immunologically ‘cold’ tumors because they largely exclude functional CTLs from the TME^7^, although they may contain small numbers of dysfunctional CTLs^8,9^. In contrast to CTLs, regulatory T cells (Treg cells) infiltrate the GBM TME, and together with M2-like macrophages/microglia constitute the predominant suppressive immune cell populations within GBMs^1,10–14^ that suppress the antitumor activities of CTLs and may mediate resistance to ICBs^15^. Furthermore, GBMs typically have highly proliferative tumor cells with low mutational burdens and neoantigen levels, limiting the ability of CTLs to recognize tumor cells and initiate an antitumor response, even after CD8 T cells are re-activated with ICBs^5,16,17^.

Glucocorticoid-induced tumor necrosis factor related protein (GITR) is an immune checkpoint that is constitutively expressed in Treg cells. GITR activation by its ligand in CD4 effector cells and CTLs increases cell proliferation and effector function. GITR stimulation in Treg cells leads to instability, Treg cell depletion, and decreases Treg cells suppressive function^18,19^. Anti-GITR agonistic antibody therapy has been shown to cause tumor regression in a number of preclinical non-CNS tumor models^18,20^ and is under evaluation as monotherapy (NCT01239134) and in combination with aPD1 therapy in a number of clinical trials (NCT04021043, NCT02740270, NCT02628574, NCT02598960, and NCT04225039). αGITR therapy combined with stereotactic radiation in an experimental model of glioma improves survival^21^. While these studies are insightful, the mechanisms mediating therapeutic response in GBM for αGITR alone or in combination with aPD1 or radiation remain to be elucidated. Specifically, effect of αGITR therapy on the GBM TME, including GBM Treg cells, is not well understood. Moreover, it is unclear whether αGITR therapy depletes or destabilizes GBM Tregs and how this therapy cooperates with αPD1 to mediate an antitumor response.

Here, using three preclinical murine models of GBM, we show that αGITR treatment converts the immunosuppressive TME of GBM into an immunostimulatory milieu by preferentially targeting GBM Treg cells. αGITR treatment converts immunosuppressive Treg cells into anti-tumor Th1-like CD4 T cells and overcomes resistance to αPD1 in experimental GBMs. This strategy has several advantages over targeting CTLs. First, since the T-cell receptor (TCR) repertoire of Treg cells is skewed toward recognizing self-antigens^22^, converted Treg cells can recognize self-antigens expressed by GBM tumor cells without requiring neoantigen epitopes as CTLs normally do. Second, unlike CTLs, Treg cells are abundantly present in the TME of GBMs; thus, strategies inducing Treg conversion to effector cells would generate large numbers of antitumor effector cells from existing GBM Treg cells that already recognize tumor self-antigens. Third, Treg cells usually are absent in healthy brain tissue^23^ but are recruited into GBMs, contributing to disease progression. Finally, the specific phenotype of GBM infiltrating Treg cells differs significantly from peripheral organs. Thus, immunotherapy targeting the specific phenotype of GBM-infiltrating Treg cells could potentially be tumor-specific without triggering systemic autoimmunity by interfering with the role of Treg cells in maintaining peripheral homeostasis. Thus, targeting Treg cells using αGITR may decrease the incidence of immune-related adverse events that often accompany systemic Treg cell depletion strategies, or CD8 CTL-targeted therapies in the clinic, which can result in treatment discontinuation and therefore loss of therapeutic benefit^24,25^. Here, we, therefore, evaluate the contribution of Treg cells to the immune TME of GBM during tumor progression and in response to ICBs, and assess whether resistance to an ICB such as αPD1 could be overcome by targeting GBM Treg cells. Finally, we evaluate if such a strategy can be effectively combined with the standard of care (SoC) therapy for newly diagnosed GBM^1^.

## Results

### αPD1 does not alter the T cell landscape in favor of GBM rejection

**Fig. 1:**
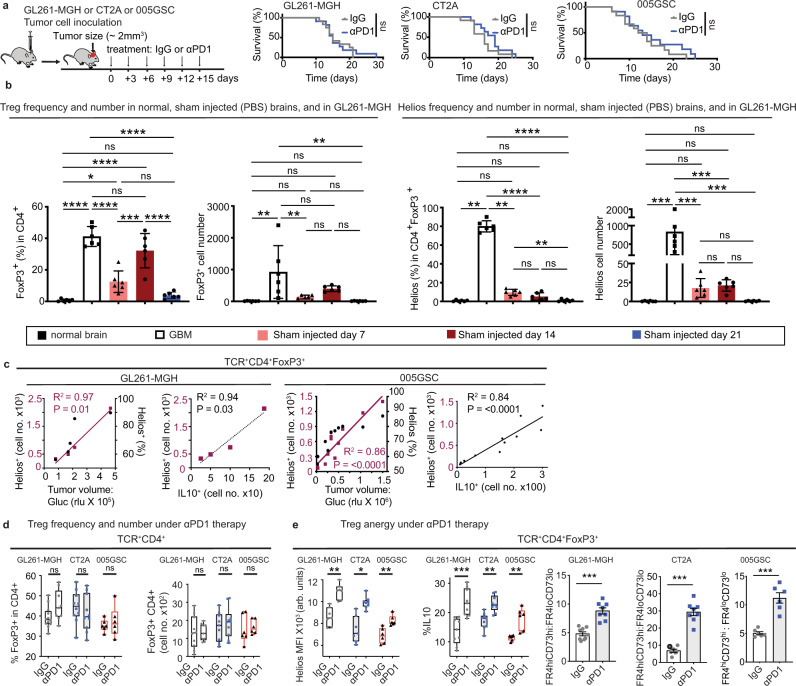
αPD1 monotherapy does not prolong GBM survival but increases Treg cell anergy in GBM tumors. **a** Schematic representation of experimental setup to evaluate the effect of IgG2a (isotype control) or αPD1 therapy on the survival of GL261-MGH (*n* = 12), CT2A (*n* = 12), and 005GSC (*n* = 13) GBM-bearing mice (two independent repeats of survival assessment). Median survival for GL261-MGH [IgG2a (15 days), αPD1 (15 days), *p* = 0.760], CT2A [IgG2a (16 days), αPD1(19 days), *p* = 0.1730], 005GSC [IgG2a (13 days), αPD1 (13 days), *p* = 0.3479]. **b** Treg cells’ frequency (% of FoxP3^+^ cells in CD4 T cells) (left) and number (second panel) in the normal brain compared to tumor tissue (GL261-MGH), and PBS injection at days 7, 14 and 21 post injection. Correlation of Helios^+^ GBM Treg cells (cell number (third panel from right) and frequency (right panel) in the normal brain, GL261-MGH tumors, and sham injected brain at days 7,14 and 21 quantified by FACS (*n* = 6 biological replicate). Data presented are mean ± SEM. *P* < 0.0001 by ordinary one-way ANOVA test and were corrected for multiple comparisons using the Tukey adjustment. For between group analysis post-Tukey, Statistical significance is shown as *p < 0.05, **p < 0.01, ***p < 0.001, ****p < 0.0001. **c** Correlation of Helios^+^ GBM Treg cells (cell number and frequency) with tumor volume (purple left *Y*-axis; black right *Y*-axis, respectively) (left for both GL261-MGH and 005GSC) and correlation of Helios expressing Treg cells with IL10 expressing Treg cells (right for both GL261-MGH and 005GSC) cell numbers in GBM) (GL261-MGH *n* = 5, 005GSC *n* = 11), using linear regression analysis. **d** Treg cell frequency(left) [GL261-MGH (IgG *n* = 12, αPD1 *n* = 9); CT2A (IgG *n* = 10, αPD1 *n* = 10); 005GSC (IgG *n* = 6, αPD1 *n* = 6)]. and cell numbers (right) [GL261-MGH (IgG *n* = 10, αPD1 *n* = 10); CT2A (IgG *n* = 10, αPD1 *n* = 10); 005GSC (IgG *n* = 6, αPD1 *n* = 6)], quantified by FACS. Data presented are mean ± SEM. *P* = 0.0011 by ordinary one-way ANOVA test and were corrected for multiple comparisons using the Tukey adjustment. For between group analysis post-Tukey, stars were assigned as *p < 0.05, **p < 0.01, ***p < 0.001. The line in the middle of the box and whisker graph represents the median (50th percentile). The box extends from the 25th to 75th percentile. The whiskers extend from the lowest to the highest value. **e** Helios expression and mean fluorescence intensity (MFI) of Helios (left) [GL261-MGH (IgG *n* = 7, αPD1 *n* = 7); CT2A (IgG *n* = 7, αPD1 *n* = 8); 005GSC (IgG *n* = 6, αPD1 *n* = 6)], percentage of IL10 positive cells (middle) [GL261-MGH (IgG *n* = 9, αPD1 *n* = 9); CT2A (IgG *n* = 9, αPD1 *n* = 9); 005GSC (IgG *n* = 6, αPD1 *n* = 6)], and the ratio of FR4^hi^CF73^hi^ to FR4^lo^CF73^lo^ in tumor infiltrating Treg cells (right) [GL261-MGH (IgG *n* = 9, αPD1 *n* = 8); CT2A (IgG *n* = 7, αPD1 *n* = 8); 005GSC (IgG *n* = 6, αPD1 *n* = 6)] quantified by FACS. Data presented are mean ± SEM. *P* < 0.0001 by ordinary one-way ANOVA test and were corrected for multiple comparisons using the Tukey adjustment. The line in the middle of the box and whisker graph represents the median (50th percentile). The box extends from the 25th to 75th percentile. The whiskers extend from the lowest to the highest value. For between group analysis of Helios MFI and IL 10 post-Tukey, stars were assigned as *p < 0.05, **p < 0.01, ***p < 0.001. Two-tailed unpaired *t*-test for the ratio of FR4^hi^CF73^hi^ to FR4^lo^CF73^lo^, ***p < 0.001 [(GL261-MGH, 95% confidence interval 2.667–5.346, *t* = 6.376, df = 15), (CT2A, 95% confidence interval 17.34–27.68, *t* = 9.412, df = 13), (005GSC, 95% confidence interval 4.092–8.259, *t* = 6.472, df = 10)]. Source data are provided as a Source Data file.

In view of heterogeneity of clinical features of human GBM, we employed three murine orthotopic tumor models—GL261-MGH, CT2A, and 005GSC—that collectively recapitulate hallmarks of GBM pathology. (These features are summarized in Table 1). Since the widely used preclinical GL261 GBM tumor model^26^ does not recapitulate the poor response to PD1 blockade seen in GBM patients^2,16^, we generated an αPD1-resistant GBM tumor model (GL261-MGH) (Fig. 1a). Because ICBs are designed to interfere with inhibitory pathways that limit T cell responses to tumors, we first determined the effect of αPD1 therapy on the T cell infiltrate in GL261-MGH. We also employed another preclinical model of GBM (CT2A) that is resistant to αPD1 and has a lower mutational burden^27^, specifically without IDH1-R132H mutation^28^ that may contribute to reduced Treg cell presence in GBM^29^. Finally, we used 005GSC, which is derived from a genetically-engineered mouse model and faithfully recapitulates the stem-like features of human GBM^30^, and thus it is likely to have a low mutational burden. Mice bearing orthotopic, size-matched murine GL261-MGH, CT2A, and 005GSC GBM tumors were treated with IgG2a control or αPD1 antibodies. In this treatment setting, we did not observe significant survival benefits (Fig. 1a), consistent with the outcomes of randomized phase III clinical trials in patients with recurrent^2^ or newly diagnosed GBM (NCT02617589)^31^. There were no significant changes in the frequency of CD8 T cells (Supplementary Fig. 1a-c), except for a modest reduction in PD1^+^Lag3^+^ exhausted CD8 T cells (Supplementary Fig. 1d). Interestingly, we observed that CD4 T cells were consistently enriched in the GBM tissues with a CD4/CD8 ratio of 2 or higher, irrespective of treatment (Supplementary Fig. 1e). A high ratio of CD4 to CD8 T cells has been reported to correlate with poor prognosis in GBM^32^, similar to the observation in a limited number of patients (*n* = 6), where two patients who survived for more than 2 years demonstrated a relatively high proportion of intratumoral CD8 T cells (Supplementary Fig. 1f). To recapitulate some recent clinical trials in GBM patients, we investigated if the addition of ICB to the SoC could alter the T cell landscape. In a cohort of 20 patients, we did not observe ICB-induced changes in the T cell landscape in favor of CD8 T cell presence (Supplementary Fig. 1g), suggesting an ICB-resistant TME in these GBMs.

**Table 1:** Clinical features of the orthotopic, immunocompetent GBM models.

Murine GBM cell line	Recapitulates features of human GBM	Response to αPD1	Mutation status	Reference
005GSC^a^	Stem-cell rich, invasive	Non-responder	IDH-wildtype	^73,82,83^
CT2A^a^	Low mutational load	Non-responder	IDH-wildtype	^27,84^
GL261-MGH^a^	Highly vascularized, necrotic, invasive	Non-responder	IDH-wildtype	^26,27,63,64,84^

^a^Orthotopic intracranial implantation through cranial window/pocket using stereotactic devise (10, 5, and 7.5 × 104 cells for GL261-MGH, CT2A, and G005 tumors, respectively). Tumor growth is monitored by ultrasound imaging or blood Gluc assay^63^.

### ICBs increase Treg cell anergy in GBM tissue and systemically

Treg cells are a specialized subset of CD4 T cells known to directly inhibit CTL function^33^. Indeed, CD4 Treg cells were present at a high frequency (>40% of CD4 T cells) and number in treatment-naive 005GSC and GL261-MGH tumors (Fig. 1b, Supplementary Fig 2a), but were absent in sections from tumor-free brain tissues (Supplementary Fig. 2a). To analyze the Treg cells associated with tumor progression vs. Treg cells recruited due to tissue injury secondary to the orthotopic injection of tumor cells into the brain, we compared time-dependent changes in Treg cell numbers and phenotype in both conditions. The frequency of Treg cells increased in a time-dependent manner and reached its maximum (~35%) at day 14 after sham injection. However, the number of Treg cells was significantly lower in sham injection compared with that in GBM (Fig. 1b). Unlike intratumoral Treg cells in GBM that increased proportionally with tumor growth (Fig. 1c), Treg cells were reduced over time and could not be detected by day 21 post sham injection-induced injury (Fig. 1b). Moreover, the phenotype of Treg cells in the brain after injury differed significantly from that in GBM tumors, as the Tregs in the former condition had limited expression of Helios compared to GBM Treg cells (Fig. 1b).

To determine whether the high frequency of Treg cells is specific to GBM or is a general characteristic of CNS tumors^34^ that contributes to their refractoriness to CTL-based therapies, we profiled the composition of T cells infiltrating GBM tumors across several mouse models of brain tumors, including primary adult GBM models (GL261-MGH, CT2A, 005GSC), pediatric brain tumor (group 3 Myc medulloblastoma), and brain metastases from breast cancer (triple-negative and ErbB2^+^) (Supplementary Fig. 2b). We found that 30–40% of tumor-infiltrating CD4 T cells were Treg cells expressing the canonical lineage-specific transcription factor FoxP3 across tested tumor models (Supplementary Fig. 2a–c), suggesting that a high frequency of Treg cells is a general characteristic of CNS tumors. Notably, the frequency of Treg cells increased with tumor size (Supplementary Fig. 2d–f). These findings are consistent with clinical data^23,35^ and suggest that GBM progression is accompanied by an influx of peripheral Treg cells into the brain malignancies.

Since the brain does not depend on Treg cells to maintain physiological homeostasis^36^, unlike peripheral tissues, we asked whether the increase in Treg cell frequency and number is also correlated with Treg cell anergy. Co-expression of CD73 and folate receptor 4 (FR4) marks anergy of CD4 T cells and indicates a loss of effector function^37,38^. We used these markers as an indication of anergy and suppressive function by Treg cells. Flow cytometry analysis also showed that Treg cells increasingly acquired an anergic phenotype with increasing tumor volume, as measured by an increased number of cells expressing the transcription factor Helios (Fig. 1c, Supplementary Fig. 2g, h) and the key immunosuppressive cytokine IL-10 (Fig. 1c). We next asked whether the lack of αPD1 therapy efficacy was associated with increased Treg cell anergy in GBM. In contrast to peripheral tumors where αPD1 therapy can lead to an increased Treg cell frequency in tumors^39^, αPD1 therapy in GBM did not significantly alter Treg cell frequency and numbers (Fig. 1d). The expression of markers of Treg cell anergy^37,40^ substantially increased with αPD1 therapy (Fig. 1e). These data are consistent with the hypothesis that Treg cells may mediate resistance to αPD1 therapy in GBM.

Despite confinement of GBMs in the intracranial space, systemic T cells are also dysfunctional in GBM patients^41^. To evaluate this dysfunction, including induction of Treg cell anergy, we analyzed tumor-draining lymph nodes and spleens of GBM tumor-bearing mice. While the frequency and numbers of Treg cells were not altered in these lymphoid organs during tumor growth (Supplementary Fig. 3a), the ratio of FR4^hi^CD73^hi^/FR4^lo^CD73^lo^ was higher compared to non-tumor-bearing mice (Supplementary Fig. 3b). These data indicate that Treg cells outside the brain can also acquire a suppressive phenotype and might induce systemic immunosuppression during tumor progression.

### Engagement of GITR converts Treg cells to T effector cells

Given the high frequency and anergy induced in GBM Treg cells in response to αPD1 treatment, we reasoned that either the depletion or the modulation of Treg cells might restore CD8 T cell-mediated antitumor immunity. We first tested the effect of Treg cell depletion by systemic administration of diphtheria toxin (DT) in GBM-bearing mice expressing the human DT receptor on Treg cells (*FoxP3*^*DTR*^)^34^, when tumors were fully established (Supplementary Fig. 4a). In contrast to peripheral tumors^42^, total ablation of Treg cells in established GBMs using DT cannot be achieved, since the required doses would cause severe morbidity. We used the maximum tolerated DT dose, which achieved a 60% reduction of Treg cell frequency in tumors (Supplementary Fig. 4b). This Treg cell reduction conferred a modest survival benefit of 3–5 days (Supplementary Fig. 4c). We did not observe a change in the frequency of CD8 T cells. We found only a modest reduction in the exhausted phenotype of CD8 T cells as demonstrated by reduced frequency of CTL receptors associated with the exhaustion (PD1, LAG-3, and TIGIT) (Supplementary Fig. 4d). In contrast, the remaining Treg cells retained their anergic phenotype (Supplementary Fig. 4e). Treatment with αPD1 did not lead to a survival benefit in DT-treated GL261-MGH bearing mice (Supplementary Fig. 4f, g). Because anti-cytotoxic T-lymphocyte associated protein (αCTLA4) treatment is known to both re-activate CTLs and deplete Treg cells^43^, we tested if combining αCTLA4 with αPD1 antibodies could alleviate Treg cell-mediated resistance to αPD1 antibody in GBM (Supplementary Fig. 5a). The combination of αPD1 and αCTLA4 antibodies did not alleviate resistance to αPD1. Specifically, the tumor burden was not reduced (Supplementary Fig. 5b), the overall survival of mice did not increase (Supplementary Fig. 5b), and the composition of tumor-infiltrating T cells did not change toward increased CTL numbers (Supplementary Fig. 5c) or function (Supplementary Fig. 5d). While αCTLA4 monotherapy did not deplete GBM-infiltrating Treg cells (Supplementary Fig. 5e), it did reduce the anergic phenotype of Treg cells (Supplementary Fig. 5f–h). However, the addition of αPD1 to αCTLA4 reversed the reduction in Treg cell anergy conferred by αCTLA4 (Supplementary Fig. 5f–h). Overall, these data indicate that the depletion of Treg cells is unlikely to yield therapeutic benefit in established GBM tumors.

We, therefore, searched for alternative approaches to specifically target tumor-infiltrating Treg cells with antibodies. We profiled GBM Treg cells for expression of co-inhibitory/activating receptors by flow cytometry (Supplementary Fig. 6), and found that GITR showed the highest expression in intratumoral GBM Treg cells, irrespective of ICB treatment (Supplementary Fig. 6a). Moreover, while GITR was highly expressed in GBM Treg cells (~85%), its expression was significantly lower in Treg cells in the spleen (Supplementary Figs. 6c, 7a) and blood (~25%) (Supplementary Fig. 6d) of tumor-bearing mice (~20%), indicating that GITR is relatively selectively expressed in GBM-infiltrating Treg cells. Furthermore, induction of GITR signaling using an agonistic GITR monoclonal antibody (αGITR here onwards) has been shown to deplete Treg cells^18,44,45^ or induce Treg cell lineage instability in non-CNS tumors^40,46^. Therefore, we reasoned that inducing GITR signaling in Treg cells may be a promising immunotherapeutic strategy to overcome resistance to ICBs in GBM as it would not only reduce suppression of antitumor immunity but also activate potential antitumor effector functions of reprogrammed Treg cells.

**Fig. 2:**
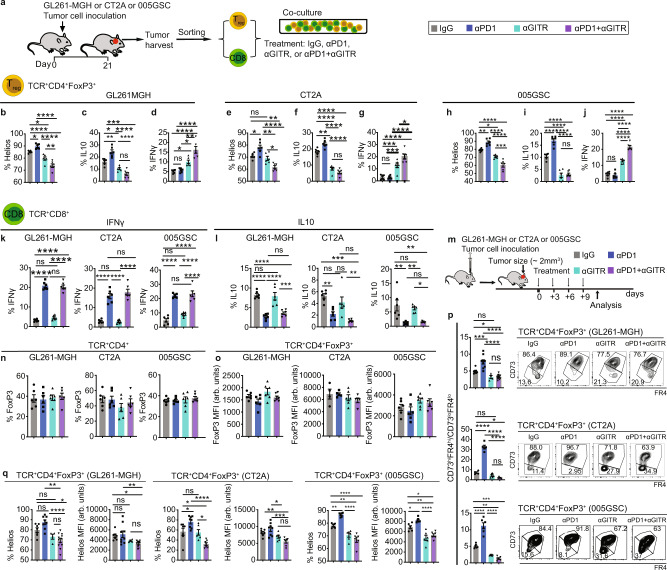
Engagement of GITR with an agonistic antibody (αGITR) converts Treg cells to CD4 T effector cells. **a** Schematic representation of the ex vivo treatment experiments of Treg cells and CD8 T cells derived from tumor-bearing mice. Tumors [GL261-MGH (*n* = 24), CT2A (*n* = 25), 005GSC (*n* = 20)] were inoculated and harvested 21 days post-inoculation. Treg cells and CD8 T cells were sorted using FACS. CD8 T cells (2 × 10^3^) were co-cultured with Treg cells (2 × 10^3^), accompanied by treatment with (i) IgG2a (isotype control), (ii) αPD1, (iii) αGITR, or (iv) αGITR + αPD1 (*n* = 6 per treatment, 1 treatment, 20 µg/mL for each antibody). **b**–**j** Treg cells (day 4) were analyzed for expression of Helios (**b**, **e**, **h**), and cytokines IL10 (**c**, **f**, **i**) and IFNγ (**d**, **g**, **j**). (*n* = 6 biological replicates for each treatment), Data presented are mean ± SEM. *P* < 0.0001 by ordinary one-way ANOVA test and were corrected for multiple comparisons using the Tukey adjustment for (**b**–**j**). Stars were assigned as *p < 0.05, **p < 0.01, ***p < 0.001, ****p < 0.0001. **k**, **l** CD8 T cells were analyzed for the production of cytokines IFNγ (K) and IL10 (L) (*n* = 6 biological replicates for each treatment). Data presented are mean ± SEM. *P* < 0.0001 by ordinary one-way ANOVA test and were corrected for multiple comparisons using the Tukey adjustment (**k** & **l**). Stars were assigned as *p < 0.05, **p < 0.01, ***p < 0.001, ****p < 0.0001. **m** Schematic representation of experimental protocol. Mice bearing orthotopic GBM tumors (GL261-MGH, CT2A or 005GSC, size ~2 mm^3^) were treated with 4 doses of: (i) IgG2a, (ii) αPD1, (iii) αGITR, or (iv) αGITR + αPD1 (250 µg/mice). **n**–**o** Intratumoral T cells were analyzed for Treg cell frequency (**n**) and FoxP3 MFI (**o**). Data presented are mean ± SEM. *P* > 0.05 by ordinary one-way ANOVA test. Treg frequency data (**n**), was a collection of 6 biological replicates per treatment arm. MFI of FoxP3 (**o**) *n* = 6 biological replicates across the tumor models and treatment arms excluding IgG treatment in CT2A (*n* = 4 biological replicates), αPD1 (*n* = 5 biological replicates) and αGITR (*n* = 7 biological replicates). Data points are not significantly different from each other (**n**–**o**). **p** Intratumoral Treg cells were analyzed for the co-expression of CD73 and FR4. GL261-MGH (IgG *n* = 7, αPD1 *n* = 9, αGITR *n* = 4, αPD1 + αGITR *n* = 11), CT2A (IgG *n* = 4, αPD1 *n* = 5, αGITR *n* = 6, αPD1 + αGITR *n* = 5), 005GSC (IgG *n* = 6, αPD1 *n* = 6, αGITR *n* = 6, αPD1 + αGITR *n* = 6). Data presented are mean ± SEM. *P* < 0.0001 by ordinary one-way ANOVA test and were corrected for multiple comparisons using the Tukey adjustment. Stars were assigned as *p < 0.05, **p < 0.01, ***p < 0.001, ****p < 0.0001. **q** Intratumoral Treg cells were analyzed for as well as expression of transcription factor Helios GL261-MGH (IgG *n* = 7, αPD1 *n* = 9, αGITR *n* = 4, αPD1 + αGITR *n* = 11), CT2A (IgG *n* = 7, αPD1 *n* = 8, αGITR *n* = 6, αPD1 + αGITR *n* = 7), 005GSC (IgG *n* = 6, αPD1 *n* = 6, αGITR *n* = 6, αPD1 + αGITR *n* = 6) and Helios MFI GL261-MGH (IgG *n* = 7, αPD1 *n* = 8, αGITR *n* = 4, αPD1 + αGITR *n* = 11), CT2A (IgG *n* = 7, αPD1 *n* = 8, αGITR *n* = 6, αPD1 + αGITR *n* = 7), 005GSC (IgG *n* = 6, αPD1 *n* = 6, αGITR *n* = 6, αPD1 + αGITR *n* = 6). Data presented are mean ± SEM. *P* < 0.0001 (GL261-MGH and 005GSC, Helios frequency, & MFI, and CT2A Helios frequency), *P* = 0.0003 (CT2A Helios MFI) by ordinary one-way ANOVA test and were corrected for multiple comparisons using the Tukey adjustment. Stars were assigned as *p < 0.05, **p < 0.01, ***p < 0.001, ****p < 0.0001. Source data are provided as a Source Data file.

Because the therapeutic effect of αGITR in ICB-responsive GL261^21^ was shown to be independent of Treg cells’ presence in GBM and largely relied on CD8 T cell gain of function, we first tested whether αGITR treatment alters the phenotype of Treg cells isolated from GL261-MGH, CT2A, or 005GSC bearing mice ex vivo. Treg cells and CD8 T cells were harvested from tumors and co-cultured in a 1:1 ratio (Fig. 2a), resembling the ratio of Treg cells to CD8 T cells in GBM (Supplementary Figs. 1e and 2b). Treg cells treated with αGITR displayed decreases in anergic phenotype in all three tumor models (Fig. 2b-j) as measured by the expression of Helios (Fig. 2b, e and h) and the production of IL-10 (Fig. 2c, f and l). Importantly, the αGITR-treated Treg cells gained CD4 effector function, as evidenced by the increased production of IFNγ^47^ (Fig. 2d, g and j). Similar to in vivo studies (Fig. 1e), αPD1 monotherapy enhanced Treg cells anergy (Fig. 2b,c, e, f and h, I). αPD1 + αGITR treated Treg cells showed a CD4 effector phenotype similar to αGITR therapy as opposed to anergic Treg cells observed in control and following αPD1 treatment. While αPD1 therapy alleviated CD8 T cell dysfunction evidenced by increased production of IFNγ (Fig. 2k) and decreased production of IL-10 (Fig. 2l), unlike immunotherapy responsive GL261 tumors^21^, αGITR monotherapy did not alter the CD8 T cell phenotype in the GL261-MGH model.

Next, we tested whether the loss of Treg cells’ anergy is preserved in vivo. Therapeutic administration of αGITR to established GBM tumors (GL261-MGH, CT2A, and 005GSC) (Fig. 2m) neither changed the Treg cell frequency (Fig. 2n) nor down-regulated FoxP3 expression (Fig. 2o). In established GBM tumors, αGITR reduced the Treg cell anergic phenotype measured by the ratio of CD73^hi^FR4^hi^ to CD73^lo^FR4^lo^ cells (Fig. 2p) and the expression of Helios (Fig. 2q). The acquired loss of anergy under αGITR persisted even when combined with αPD1 that normally induces an anergic phenotype in Treg cells (Fig. 2p, q).

We then tested whether αGITR treatment induces loss of the suppressive phenotype of Treg cells in vivo. We treated GL261-MGH-bearing mice with 4 doses of (i) isotype control (IgG2a), (ii) αPD1 antibody, (iii) αGITR antibody, or (iv) αPD1 + αGITR antibodies. We then isolated Treg cells from LNs and spleens, and co-cultured them with tumor-naive CD8 T cells harvested from spleens of healthy mice (Supplementary Fig. 8a). We reasoned that while intratumoral Treg cells may differ significantly from peripheral Treg cells (Supplementary Fig. 6, 7), they share certain similarities in anergic phenotype, including the increased co-expression of CD73 and FR4 (Supplementary Fig. 3). We found that in contrast to Treg cells from mice treated with IgG2a or αPD1 alone, Treg cells from mice treated with αGITR antibody or combined αPD1 + αGITR antibodies (Supplementary Fig. 8a) lost their ability to suppress CD8 T cell proliferation (Supplementary Fig. 8b), which increased the number of IFNγ-secreting CD8 T cells (Supplementary Fig. 8c).

Finally, we asked whether αGITR treatment could also impact CD8 T cells’ ability to resist Treg cell suppression using the same treatment groups as above (Supplementary Fig. 8d). We found that, in contrast to CD8 T cells from mice treated with IgG2a or αPD1 alone, CD8 T cells harvested from mice treated with αGITR alone or combined αPD1 + αGITR gained an ability to resist Treg cell suppression and therefore were significantly more proliferative and exhibited increased IFNγ expression (Supplementary Fig. 8e, f).

To test whether αGITR-treated Treg is sufficient to overcome immune suppression by residual Treg cells during genetic depletion, we treated GL261-MGH tumor-bearing FoxP3^*DTR*^ mice with either DT (to partially deplete Treg cells) alone or DT + αGITR (to partially deplete and reprogram the remaining ones) (Supplementary Fig. 9a). We observed that αGITR treatment alone or αGITR following partial Treg cell depletion led to a significant survival benefit compared with control (Supplementary Fig. 9b). Moreover, αGITR treatment, along with Treg cell depletion, also showed a trend (*p* = 0.051) toward prolonged survival of GBM-bearing mice over Treg cell depletion alone (Supplementary Fig. 9b).

Because of the prevalence of tumor-associated macrophages (TAMs) in GBM^10,29^, we also analyzed the effect of Treg cell depletion on the frequency and phenotype of TAMs. Partial Treg cell depletion using the DTR mouse model increased the fraction of “M1-like” over “M2-like” TAMs, although it did not reduce the frequency of TAMs (Supplementary Fig. 10a). Since TAMs are known to regulate CD4 T cells in GBM^10^, we asked the relative contribution of Treg cells vs. TAMs in GBM tumor development. To this end, we compared the GBM take rate in mice depleted of Treg cells (*FoxP3*^DTR^ mice, treated with DT prior to tumor inoculation) vs. *Ccr2*^–/–^ mice that are unable to recruit monocytic TAM precursors to tumor sites (Supplementary Fig. 10b). CCR2 expression is not restricted to monocytes but is also high in microglia^48^, and its deficiency impairs the accumulation of both microglia and macrophages^48^. Importantly, fate mapping using *CCR2-CreER* mice showed monocyte to microglia transition during development and in response to injury^49^. Because of these reasons, we selected *Ccr2*^−/−^ mice rather than *Cx3cr1*^−/−^ mice to be able to include all macrophage subsets that are involved in shaping myeloid immune microenvironment in this particular tumor type. In mice depleted of Treg cells, 30% of them rejected GBM tumors (3 out of 10). In contrast, the tumor take rate in *Ccr2*^–/–^ mice was not significantly changed, compared to wild-type mice (Supplementary Fig. 10b). Furthermore, because a positive feedback loop between Treg cells and TAMs has been described in non-CNS tumors^50^, we generated *FoxP3*^DTR^*Ccr2*^–/–^ mice to investigate whether the reduction of both Treg cells and TAMs could restore antitumor immunity in GBM. We did not observe significant improvement in tumor growth or survival in DT-treated *FoxP3*^*DTR*^*Ccr2*^–/–^ mice beyond that seen in *Ccr2*^–/–^ mice or *FoxP3*^*DTR*^ mice treated with DT (Supplementary Fig. 10c). To evaluate whether a combination of reducing and converting Treg cells, and reducing TAMs can alleviate resistance to αPD1 therapy, we treated *FoxP3*^*DTR*^*Ccr2*^–/–^ mice bearing GBMs (GL261-MGH and CT2A) with DT + αGITR + αPD1 in a 6-arm experiment and monitored mouse survival. We found that modulation of Treg cells (depletion + conversion) along with the reduction of TAMs enhanced the survival in both GL261-MGH (~2-fold increase) and CT2A (~3.5-fold) challenged mice (Supplementary Fig. 10d). Importantly, this therapy resulted in total tumor eradication in ~35% of CT2A-bearing mice (Supplementary Fig. 10d). Altogether, these data suggest that Treg cells contribute critically to immunosuppression in conjunction with TAMs in GBM, despite the former being present in relatively lower numbers than TAMs.

### αPD1 + αGITR convert GBM Treg cells to Th1 effector T cells

**Fig. 3:**
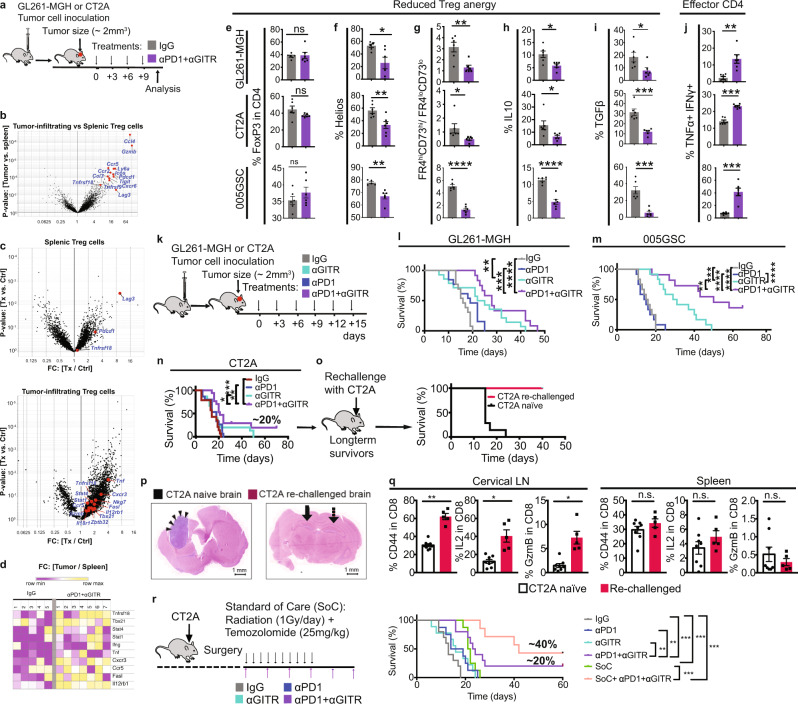
αPD1 + αGITR converts GBM Treg cells to Th1 effector T cells leading to enhanced survival and CD8 T cell memory phenotype in mice. **a** Schematic representation of experimental protocol, where mice bearing orthotopic GL261-MGH, CT2A, or 005GSC tumors and treated with 4 doses of either IgG2a (isotype control), or αGITR + αPD1 (*n* = 6, day 10 post-randomization and treatment). Spleen and tumors were harvested, and cells were sorted for Treg cells. **b**–**d** RNA-seq analysis was performed on GBM Treg cells and spleens of IgG2a (isotype control, *n* = 5) vs. αGITR + αPD1 treated CT2A bearing animals (*n* = 7). **b** Volcano plots showing differentially expressed genes (DEGs) between spleen and GBM Treg cells treated with IgG2a. **c** Volcano plot showing DEGs between Treg cells from mice treated with IgG2a or αGITR + αPD1 in the spleen (Top panel) and GBM (lower panel). Genes of interest are highlighted. **d** Heatmap of representative Th1 genes expressed in GBM Treg cells upon IgG2a (left) or αGITR + αPD1 (right) treatment. **e**–**j** Intratumoral Treg cells in mice bearing orthotopic GL261-MGH, CT2A, or 005GSC tumors and treated with 4 doses of either IgG2a (isotype control) or αGITR + αPD1 (*n* = 6 biological replicate, day 10 post-randomization and treatment) analyzed by flow cytometry for (**e**) Treg cell frequency [(GL261-MGH, *P* = 0.8819), (CT2A, *P* = 0.1108), (005GSC, *P* = 0.2753)] (**f**) expression of Helios [(GL261-MGH, *P* = 0.0173, 95% confidence interval:−48.63 to −5.949), (CT2A, *P* = 0.009, 95% confidence interval:), (005GSC, *P* = 0.0016, confidence interval:−16.41 to −5.383)], (**g**) co-expression of CD73 and FR4 [(GL261-MGH, *P* = 0.0034, 95% confidence interval: −2.930 to −0.7693), (CT2A, *P* = 0.0455, 95% confidence interval: −38.89 to −7.140), (005GSC, *p* < 0.001, confidence interval:−4.544 to −2.939)], (**h**) production of IL-10 [(GL261-MGH, *P* = 0.0108, 95% confidence interval: −7.707 to −1.293), (CT2A, *P* = 0.0329, 95% confidence interval: −17.11 to −0.8946), (005GSC, *p* < 0.001, confidence interval: −8.447 to −4.595)], (**i**) TGFβ [(GL261-MGH, *P* = 0.0362, 95% confidence interval: −20.59 to −0.8395), (CT2A, *P* = 0.0002, 95% confidence interval: −27.18 to −11.58), (005GSC, *P* = 0.0002, confidence interval:−37.65 to −16.17)], and (**j**) coproduction of IFNγ and TNFα [(GL261-MGH, *P* = 0, 95% confidence interval: 0.2742–19.22), (CT2A, *p* < 0.001, 95% confidence interval: 6.848–11.15), (005GSC, *P* = 0.0002, confidence interval: 20.87–47.81)]. Two-tailed unpaired *t*-test for these analyses, and stars were assigned as *p < 0.05, **p < 0.01, ***p < 0.001, ****p < 0.0001. **k** Schematic representation of the experimental protocol. **l**–**n** Mice bearing orthotopic GBM tumors (GL261-MGH, 005GSC or CT2A, size ~2 mm^3^) were treated with six doses of (i) IgG2a, (ii) αPD1, (iii) αGITR, or (iv) αGITR + αPD1 (250 µg/mice for each antibody) and monitored for survival, (Survival study repeated two times). GL261-MGH [(IgG *n* = 13 and median survival 16 days), (αPD1 *n* = 13 and median survival 19 days), (αGITR *n* = 14 and median survival 23.5 days), (αPD1 + αGITR *n* = 12 median survival 28 days)], CT2A ([(IgG *n* = 14 and median survival 14 days), (αPD1 *n* = 15 and median survival 18 days), (αGITR *n* = 15 and median survival 18 days), (αPD1 + αGITR *n* = 13 median survival 21 days), 005GSC ([(IgG *n* = 12 and median survival 15.5 days), (αPD1 *n* = 12 and median survival 14.5 days), (αGITR *n* = 12 and median survival 30 days), (αPD1 + αGITR *n* = 11 median survival 50 days). Statistical significance is shown as *p < 0.05, **p < 0.01, ***p < 0.001, ****p < 0.0001. **o** Long-term survivors (4 out of 24 mice) with no evidence of residual CT2A tumors in the CT2A cohort were re-challenged with CT2A cells in the contralateral hemisphere of the brain and compared with CT2A naïve mice (*n* = 7) that were inoculated with CT2A tumor. Survival data is representative of 2 independent experiments. CT2A naïve (median survival of 15 days), and CT2A-rechallenged (not applicable). **p** Arrows indicate the tumor inoculation region in the brain. Histology of brains from tumor-bearing brains (left) where arrowheads indicate a tumor, and CT2A re-challenged mice (right) where the arrow indicates the primary injection site, and the dashed arrow indicates the secondary injection site. **q** Levels of activation and cytokine production by CD8 T cells from spleen and cervical LNs of re-challenged mice (*n* = 5) and CT2A naïve mice (*n* = 8, day 12 post-randomization). Two-tailed unpaired *t*-test for these analyses, and stars were assigned as *p < 0.05, **p < 0.01, ***p < 0.001, ****p < 0.0001. Draining Lymph node: [(%CD44 in CD8, *p* < 0.0001, 95% confidence interval: 24.38–39.23), (%IL2 in CD8, *P* = 0.0006, 95% confidence interval: 15.22–41.17), (%GzmB in CD8, *p* = 0.0004, confidence interval: 3.282–8.318)] and Spleen: [(%CD44 in CD8, *P* = 0.2537), (%IL2 in CD8, *P* = 0.2538), (%GzmB in CD8, *p* = 0.4357)]. **r** Schematic representation of experimental protocol where mice bearing orthotopic CT2A tumors received tumor resection, followed by treatment with IgG2a (control, *n* = 8), αPD1 (*n* = 8), αGITR (*n* = 9), αGITR + αPD1 (*n* = 8), clinically relevant standard of care (SoC, *n* = 8) consisting of cranial radiation therapy (1 Gy per day for 10 days) and concomitant daily chemotherapy with temozolomide 25 mg (i.p. for 10 days) or combination treatment with surgery, or chemo-radiation plus αGITR + αPD1 therapy (*n* = 7). Kaplan–Meier survival estimates were compared using the Mantel–Cox log-rank test as well as the Gehan–Breslow–Wilcoxon test. *P* values for tumor viability were calculated using unpaired *t*-tests. *p < 0.05, **p < 0.01, ***p < 0.001. CT2A ([(IgG median survival 8 days), (αPD1 median survival 9 days), (αGITR median survival 22.5 days), (αPD1 + αGITR median survival 21.5 days), (SoC median survival 14 days), (SoC+αGITR + αPD1 median survival 42 days)]. Source data are provided as a Source Data file.

To gain mechanistic insights into αGITR-treated Treg cells and to identify the transcriptional networks involved, we performed RNA-seq analysis using splenic and intratumoral Treg cells from GBM-bearing mice treated with IgG2a (isotype control) vs. αPD1 + αGITR (Fig. 3a). We focused on the αPD1 + αGITR combination therapy arm rather than the monotherapy arms because Treg cell conversion that occurred under αGITR therapy ex vivo and in vivo was extended to dual therapy (Fig. 2). We also observed that αPD1 therapy predominantly affects CD8 T cell function (Fig. 2k, l) rather than CD4 Treg cells. Our analysis revealed that co-stimulatory genes such as *Lag3*, *Pdcd1* (which encodes PD1), *Icos*, *Tnfrsf9* (which encodes 4-1BB), *Tigit*, and *Tnfrsf18* (which encodes GITR) were expressed at significantly higher levels in the GBM Treg cells compared to the splenic Treg cells (Fig. 3b). In comparison to 155 significantly differentially expressed genes (DEGs; FC > 2, *P* < 0.05) in splenic Treg cells, we found 744 DEGs in GBM-infiltrating Treg cells after αPD1 + αGITR therapy (Fig. 3c), suggesting αGITR treatment primarily modulated GBM Treg cells. Further analysis demonstrated that GBM Treg cells acquired a Th1-like signature, including increased *Tbx21, Stat4, and IL12rb1* expression^51^, which may have contributed to de novo acquisition of antitumor function (Fig. 3c, d). Consistent with the upregulation of genes associated with the Th1-like phenotype by GBM Treg cells, treatment with αPD1 + αGITR in mice displayed reduced Treg cell anergy (Fig. 3e–g), including decreased production of TGFβ and IL-10 (Fig. 3h, i) and increased Treg cell fraction secreting the effector cytokines IFNγ and TNFα (Fig. 3j).

αPD1 + αGITR, but not αPD1 monotherapy, reduced Treg-mediated suppression of CD8 T cells ex vivo (Supplementary Fig. 8) and in vivo, as shown with the overall profiles of CD8 T cells (Supplementary Fig. 11) in co-expression of receptors that are associated with CTLs exhaustion^52^. Thus, we next tested the efficacy of αPD1 + αGITR therapy in GBM-bearing mice (Fig. 3k). Combined αPD1 + αGITR treatment significantly increased the median survival of GL261-MGH, 005GSC, and CT2A-bearing mice by 1.75-, 3.3-, and 1.5-fold, respectively, while αPD1 treatment alone did not confer any therapeutic benefit (Fig. 3l–n). Importantly, αPD1 + αGITR therapy resulted in tumor eradication in ~40% of mice bearing 005GSC tumors. Moreover, ~20% of mice with CT2A tumors (4/24 in one experiment and similar ratios in others) in the αPD1 + αGITR cohort showed a complete response and were long-term, disease-free survivors at the end of the treatment course, extending to 30 days after completion of the treatment (Fig. 3o).

The observation that there were long-term disease-free survivors in the CT2A-challenged group that received the combination treatment prompted us to test whether the long-term survivors had developed memory responses. T cell memory formation is a hallmark of the durability of a therapeutic response due to protective immunity, and thus it is a good clinical correlate of overall survival^53,54^. We re-challenged the animals that were cured of CT2A tumors by orthotopically implanting new CT2A tumors into the contralateral brain hemisphere. All re-challenged mice rejected a second tumor inoculation (Fig. 3o), and no evidence of tumors was found in brain histological sections at 6–8 weeks after re-challenge (Fig. 3p). We also analyzed the memory T cell responses in dLNs and spleens of re-challenged mice 60 days after tumor re-implantation and compared them with mice bearing primary CT2A tumors. CD8 T cells isolated from cervical draining LN of re-challenged animals contained higher frequencies of cells expressing the activation marker CD44, and also had an increased proportion of cells secreting CD8 effector molecules IL-2 and Granzyme B (GzmB) (Fig. 3q). This memory response was restricted to the cervical dLN as it was not observed in the spleen (Fig. 3q), indicating that a local but not a systemic memory response had developed in the CT2A model after αPD1 + αGITR treatment.

Because GBMs are treated with the SoC, including resection of GBM tumors to reduce the tumor burden followed by chemotherapy (temozolomide) and radiation therapy^55^, we asked whether αPD1 + αGITR can synergize with the SoC. We found that SoC therapy alone did not improve the survival of mice beyond αPD1 + αGITR or induce long-term survivors. Combining SoC with αPD1 + αGITR therapy, however, significantly increased the lifespan of tumor-bearing mice and increased the fraction of the long-term survivors compared to αPD1 + αGITR in CT2A-bearing mice (Fig. 3r).

### Treg cells are sufficient to elicit therapeutic effects of αPD1 + αGITR

**Fig. 4:**
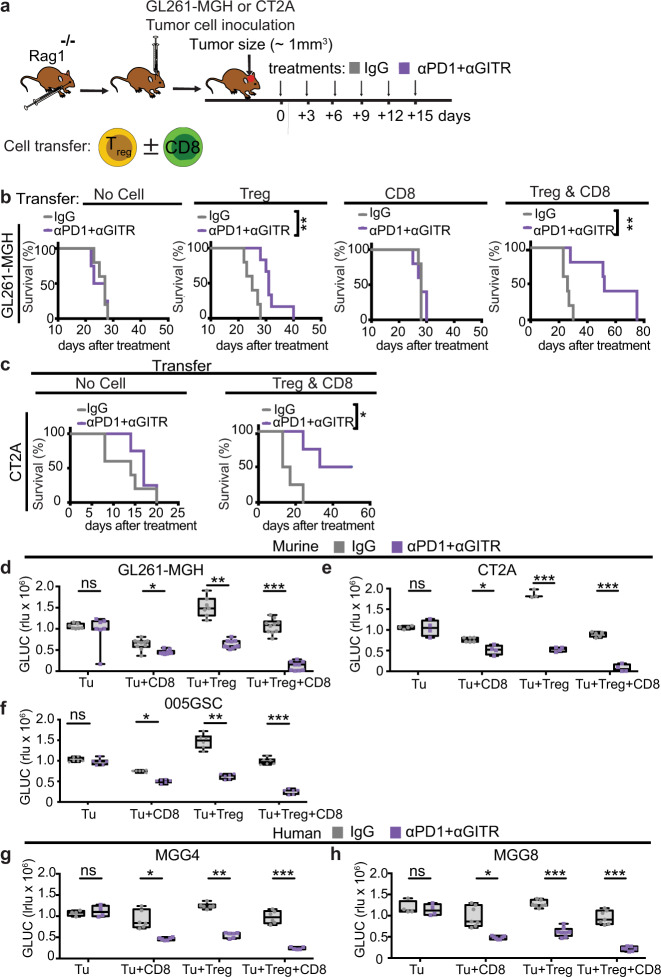
CD4 Treg cells are sufficient to elicit the therapeutic effect of αPD1 + αGITR and induce tumor cell killing. **a** Schematic representation of the experimental setup to evaluate the contributions of CD8 T cells and Treg cells to antitumor activity against GBM during αPD1 + αGITR treatment. Purified and sorted CD8 T cells (1 × 10^6^), Treg cells (5 × 10^5^), or both were transferred to *Rag1*^−/−^ mice 2 days before intracranial inoculation with tumor cells (GL261-MGH or CT2A). **b**–**c** GL261-MGH and CT2A bearing mice were treated with 6 doses of IgG2a or αPD1 + αGITR (250 μg/mouse for each antibody) and monitored for survival. [(GL261-MGH no cell: *n* = 5, IgG median survival of 27; αPD1 + αGITR, *n* = 5 median survival of 25], [(GL261-MGH CD8 T cells: IgG *n* = 5 median survival of 27; αPD1 + αGITR, *n* = 5 median survival of 25], [(GL261-MGH Treg cells: IgG *n* = 5 median survival of 25; αPD1 + αGITR, *n* = 6 median survival of 31], [(GL261-MGH CD8 + Treg cells: IgG *n* = 5 median survival of 26; αPD1 + αGITR, *n* = 5 median survival of 52]; [(CT2A no cell: *n* = 5, IgG median survival of 14; αPD1 + αGITR, *n* = 4 median survival of 17], [(CT2A CD8 + Treg cells: IgG *n* = 4 median survival of 15; αPD1 + αGITR, *n* = 4 median survival undefined]. Kaplan–Meier survival estimates were compared using the Mantel–Cox log-rank test as well as the Gehan–Breslow–Wilcoxon test. *P* values for tumor viability were calculated using unpaired *t*-tests. *p < 0.05, **p < 0.01. **d**–**h** Treg cells and CD8 T cells were isolated from spleens of healthy donor mice (*n* = 6 per condition) or human peripheral blood mononuclear cells (pooled from *n* = 3 donors). **d**–**f** Murine GL261-MGH or CT2A or 005GSC (**g**–**h**) human MGG4 or MGG8 tumor cells (1 × 10^3^ cells) were co-cultured for 48 h with Treg cells, CD8 T cells, or both in ratios of 1:10. GLUC activity in the cell culture media was measured and used as a surrogate marker for tumor cell viability. Tumor cell viability was measured under treatment with IgG2a (isotype control) or αPD1 + αGITR. Data presented are mean ± SEM. *p* < 0.001 by ordinary one-way ANOVA test and were corrected for multiple comparisons using the Tukey adjustment. For between group analysis post-Tukey, stars were assigned as *p < 0.05, **p < 0.01, ***p < 0.001. The line in the middle of the box and whisker graph represents the median (50th percentile). The box extends from the 25 to 75th percentile. The whiskers extend from the lowest to the highest value. Source data are provided as a Source Data file.

To define the major cell subsets that contribute to the therapeutic effects of αPD + αGITR therapy, we adoptively transferred Treg cells, CD8 T cells, or both Treg cells and CD8 T cells into *Rag1*^–/–^ hosts, followed by orthotopic implantation of GL261-MGH or CT2A GBM tumors. After tumors were established (~1 mm^3^), mice were subsequently treated with control IgG2a or αPD1 + αGITR by randomization into groups (Fig. 4a, Supplementary Fig. 12a). αPD1 + αGITR treatment did not result in a significant delay of GL261-MGH tumor growth in mice without or with CD8 T cell transfer. However, mice that received Treg cells or Treg cells plus CD8 T cells showed a significant reduction of tumor growth and prolonged survival after αPD1 + αGITR treatment compared to control IgG2a treatment in GL261-MGH tumors (Fig. 4b, Supplementary Fig. 12b). These beneficial therapeutic effects of αPD1 + αGITR were also observed in *Rag1*^–/–^ mice after adoptive transfer of Treg cells plus CD8 T cells followed by orthotopic inoculation with CT2A tumor cells (Fig. 4c, Supplementary Fig. 12c). Although the presence of both Treg cells and CD8 T cells showed increased survival benefit upon αGITR + αPD1 treatment, we found that Treg cells reconstitution alone was sufficient to elicit significant tumor growth delay and animal survival (Fig. 4b).

We next sought to define the mechanism by which reprogrammed Treg cells cooperate with CD8 T cells to eliminate GBM tumor cells. We first investigated the anti-GBM cytotoxicity of Treg cells, CD8 T cells, or both, by co-culturing them with GBM tumor cells in the presence of αPD1 + αGITR or control IgG2a antibodies. We found that a combination of CD8 T cells and Treg cells reprogrammed with αPD1 + αGITR was most effective for killing tumor cells (Fig. 4d-f and Supplementary Fig. 13a). Neither αPD1 nor αGITR antibodies had direct cytotoxic effects on tumor cells in our cytotoxicity assay (Supplementary Fig. 13b). We also obtained similar results in experiments testing the cytotoxicity of CD8 T cells and Treg cells derived from human peripheral blood mononuclear cells (PBMCs) against human GBM tumor cells—MGG4 and MGG8 that are known to recapitulate human GBM features^56^—in the presence of αPD1 + αGITR antibodies (Fig. 4g, h). These observations indicate that reprogrammed Treg cells gain cytolytic activity against tumor cells in addition to losing their suppressive activity on CD8 T cell proliferation and function.

### Anti-GBM effects require MHC recognition and IFNγ-mediated cytolysis

Because GBMs are known to be poorly immunogenic due to the downregulation of major histocompatibility complex (MHC) molecules (class I and II)^57^, we sought to determine the mechanism of MHC induction on GBM tumors, rendering them susceptible to T cell cytotoxicity. We previously observed that both αGITR treated Treg cells and CD8 T cells produced the effector cytokine IFNγ (Fig. 2, Fig. 3, Supplementary Fig. 8), which upregulates surface MHC expression^58^. Thus, we hypothesized that T cell-derived IFNγ might induce GBM tumors to increase surface MHC expression. Indeed, GL261-MGH and CT2A tumor cells upregulated both MHC I and II in response to IFNγ stimulation in vitro in a dose-dependent manner (Supplementary Fig. 14). Furthermore, blockade of IFNγ with a neutralizing antibody abrogated the ability of T cells to kill tumor cells (Supplementary Fig. 15, Supplementary Fig. 16), suggesting the role of T cell-derived IFNγ in the regulation of MHC molecules and therapeutic responses to αGITR + αPD1 potentially through direct recognition of antigens.

**Fig. 5:**
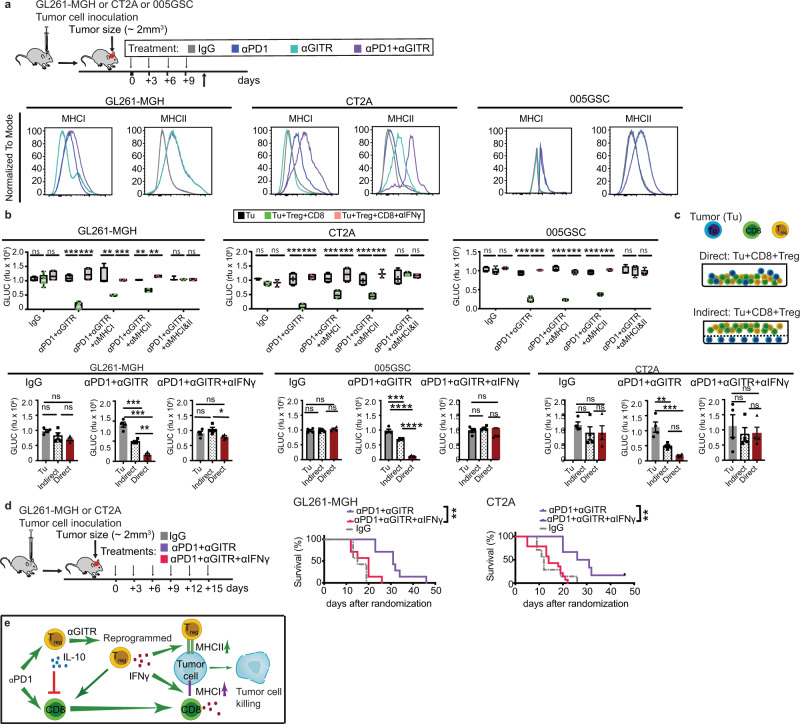
MHC dependent recognition and IFNγ-mediated cytolysis are required for antitumor activity of reprogrammed Treg cells and re-activated CD8 T cells. **a** Schematic representation of the experimental protocol. Mice bearing orthotopic GBM tumors (GL261-MGH, 005GSC or CT2A, size ~2 mm^3^) were treated with 4 doses of (i) IgG2a, (ii) αPD1, (iii) αGITR, or (iv) αGITR + αPD1 (250 µg/mice for each antibody), and tumor cells were analyzed for expression of MHCI and MHCII by FACS (*n* = 6). Histograms represent the expression level of each molecule after therapy. **b** Treg cells and CD8 T cells were sorted from spleens of healthy donor mice (*n* = 6 per condition). GL261-MGH, CT2A, or 005GSC tumor cells (1 × 10^3^ cells) were co-cultured with both Treg cells and CD8 T cells at a 1:10 ratio for 48 h. GLUC activity in the cell culture media was measured and used as a surrogate marker for tumor cell viability. Tumor cell viability was measured under treatment with IgG2A (isotype control) or αPD1 + αGITR. Impact of the blockade of MHC Class I, MHC Class II, combined MHC Class I & II, and IFNγ on the efficacy of αPD1 + αGITR treatment was analyzed. Data presented are mean ± SEM. *p* < 0.001 by ordinary one-way ANOVA test and were corrected for multiple comparisons using the Tukey adjustment. For between group analysis post-Tukey, stars were assigned as *p < 0.05, **p < 0.01, ***p < 0.001. The line in the middle of the box and whisker graph represents the median (50th percentile). The box extends from the 25 to 75th percentile. The whiskers extend from the lowest to the highest value. **c** Evaluation of the contribution of T cell-derived IFNγ to tumor cell killing. GL261-MGH, CT2A or 005GSC tumor cells were co-cultured for 48 h with Treg cells and CD8 T cells at a ratio of 1 tumor cell to 5T cells, either in direct contact or indirect contact in separate chambers of a transwell plate. Tumor cell viability was measured by measuring GLUC activity in cell culture media as in (**b**) (*n* = 4 biological replicates for all treatments). Data presented are mean ± SEM. p < 0.001 (GL261-MGH and 005GSC) and *p* = 0.0004 (CT2A) for αPD1 + αGITR therapy by ordinary one-way ANOVA test and were corrected for multiple comparisons using the Tukey adjustment. Other treatments (IgG or αPD1 + αGITR + αIFNγ) by one-way ANOVA test were above 0.05 (insignificant). **d** Schematic of the experimental setup to evaluate the contribution of IFNγ to anti-tumor T cell activity in vivo. Mice bearing orthotopic GBM tumors (GL261-MGH or CT2A, size ~2 mm^3^) were treated with 6 doses of (i) αPD1 + αGITR, and (iv) αPD1 + αGITR + αIFNγ (250 μg/mouse). [(GL261-MGH: *n* = 7, IgG median survival of 15; αPD1 + αGITR *n* = 7 median survival of 31; and αPD1 + αGITR + αIFNγ *n* = 7, median survival of 20], [(CT2A: *n* = 14, IgG median survival of 14; αPD1 + αGITR *n* = 5 median survival of 28.5; and αPD1 + αGITR + αIFNγ *n* = 7, median survival of 12]. Kaplan–Meier survival estimates in (**d**) were compared using the Mantel–Cox log-rank test as well as the Gehan–Breslow–Wilcoxon test. *p < 0.05, **p < 0.01, ***p < 0.001. **e** Schematic representation of the identified mechanism: converted Treg cells and re-activated CD8 T cells post αPD1 + αGITR treatment cooperate in activity, produce IFNγ that induces MHC upregulation on tumor cells allowing for recognition and tumor cell killing. Source data are provided as a Source Data file.

To evaluate whether αGITR, αPD1, or αGITR + αPD1 therapy induces MHC molecules in vivo, we treated GL261-MGH, CT2A, and 005GSC-bearing mice with indicated treatments and measured MHC expression on tumor cells. In line with our in vitro findings, αPD1 and αGITR selectively upregulated MHC I and MHC II, respectively. Together, αGITR + αPD1 upregulated both MHC I & II molecules on tumor cells (Fig. 5a). Next, to test whether CD8 T cells and CD4 Treg cells recognize and kill target cells via MHC I and II, respectively, we tested the contributions of each T cell subset to antitumor cytotoxicity in the presence or absence of blocking antibodies for MHC I and II. As expected, MHC I blockade abrogated the antitumor effect of CD8 T cells, while blocking MHC II compromised the antitumor effect of converted Treg cells (Fig. 5b). Blockade of both MHC I and MHC II eliminated all antitumor effects imparted by CD8 T cells and converted Treg cells (Supplementary Fig. 15, Supplementary Fig. 16), suggesting that direct TCR-MHC interaction is required for antitumor effects and that both arms of the T cell response jointly contribute to tumor killing.

Since we observed that the maximum tumor cell killing effect in mice and human is through the cooperation of converted Treg cells and CD8 T cells, we asked whether direct cell contact is required for tumor cell killing. Thus, we compared the viability of GBM cells in direct contact with Treg + CD8 T cells or GBM cells separated from Treg + CD8 T cells by a transwell membrane. While we observed reduced tumor cell viability in the transwell setup, the tumor killing ability was significantly lower than when T cells were in direct contact with tumor cells (Fig. 5c), suggesting that optimal T cell-mediated killing of GBM tumors is contact-dependent. We also confirmed that IFNγ was required for killing of the tumor cells, as we observed no antitumor cytotoxicity upon IFNγ blockade in the transwell assays (Fig. 5c). Importantly, in vivo blockade of IFNγ abrogated the antitumor effect of αGITR + αPD1 in mice (Fig. 5d), confirming the IFNγ-mediated therapeutic response to αPD1 + αGITR.

We also tested whether the cytotoxic activity by Treg cells and CD8 T cells upon αPD1 + αGITR treatment was dependent on GzmB and TNFα, in addition to IFNγ. Neither GzmB blockade nor TNFα blockade was able to abrogate the therapeutic efficacy of αPD1 + αGITR in both mouse and human cell settings (Supplementary Fig. 17, Supplementary Fig. 18). GzmB blockade abrogated CD8 T cell tumoricidal activity, but converted Treg cells maintained their ability to kill tumor cells (Supplementary Fig. 17, Supplementary Fig. 18). The lack of impact of the GzmB blockade suggests that αGITR -treatment has altered Treg cells’ fundamental function, as they are known to exert immune suppression in part through GzmB-mediated killing of target cells^59^.

Because CD4 T effector cells with tumor-killing capacity are known to preferentially lyse targets via the Fas/Fas ligand (FasL) interaction^60^, we tested whether the Fas/FasL axis is involved in converted Treg cell-mediated tumor killing by blocking the Fas receptor with an anti-Fas antibody. Fas blockade did not significantly reduce the cytotoxicity of Treg cells reprogrammed with αPD1 + αGITR (Supplementary Fig. 15), excluding the role of Fas ligation in converted Treg cell-mediated tumoricidal activity.

Taken together, these data show that αGITR treatment reprograms the immunosuppressive TME of GBM into an immunostimulatory milieu by preferentially targeting GBM Treg cells. αGITR treatment converts immunosuppressive Treg cells into anti-tumor Th1-like CD4 T cells and cooperate with CD8 T cells in killing GBM tumor cells through TCR-MHC engagement-dependent mechanisms. T cell-derived IFNγ plays a critical role in the antitumor activity of αGITR-converted Treg cells. Mechanistically, T cell-derived IFNγ increased the susceptibility of tumor cells to T cell recognition and elimination via upregulation of MHC molecules and IFNγ-mediated tumoricidal activity (Fig. 5e)^61^.

## Discussion

While the mechanisms of cancer resistance to immunotherapy are clearly multifactorial, the TME plays a critical role in the resistance of GBM to ICBs. The main immune cell types thought to be responsible for the generation of an immunosuppressive TME in most tumors are TAMs and CD4 Treg cells^62^. TAMs constitute the most abundant immune cell population in GBM and have been the target of concerted research efforts to alleviate immunosuppression in human GBM tumors^63,64^. Here, we found, however, that near-total depletion of Treg cells but not suppression of macrophage recruitment into the TME significantly reduced the tumor take rate in orthotopic mouse models of GBM (Supplementary Fig. 10). Moreover, partial depletion of Treg cells, coupled with the exclusion of macrophages (the dominant immune cells present in GBM), did not further improve survival over partial Treg cell depletion alone (Supplementary Fig. 10). We, therefore, focused our study primarily on Treg cell-mediated immunosuppression and targeting the Treg cell population for GBM immunotherapy.

Upon αPD1 treatment, Treg cells not only suppress CTL function (Supplementary Fig. 8) but also acquire an anergic phenotype (CD73^hi^FR4^hi^, Helios^hi^, and IL-10^hi^) (Figs. 1–3, Supplementary Fig. 5f-h). These data demonstrate the impact of Treg cells on generating an immunosuppressive TME that is resistant to conventional ICB therapy (e.g., αPD1 with and without αCTLA4) in GBM. This data is in contrast to recent findings that have shown Treg cell-independent resistance to αPD1 therapy in GBM^10^ and that therapeutic responses rely on CD8 and conventional CD4 T cells. This discrepancy may be due to the use of an immunotherapy responsive or partially responsive GL261 tumor model. By contrast, we developed a variant of GL261 (GL261-MGH) that resists αPD1 and mimics patient outcomes. Our data, however, is in line with chimeric antigen receptor (CAR) T cell refractoriness to therapy. GBM tumors in patients that relapse from CAR T cell therapy are invaded with a large number of Treg cells^65^. Collectively, these findings demonstrate the importance of mimicking the TME in mice that resembles patient outcomes to understand the tumor development, therapeutic response, and potential resistance to therapy.

To alleviate Treg cell-mediated resistance, strategies that rely on targeting Treg cells should be restricted to the tumor site since Treg cells are required for the maintenance of immune homeostasis and self-tolerance that prevents the development of autoimmune diseases^40,66^. GITR represents an ideal targetable surface immunomodulatory receptor because of its high levels of expression on GBM Treg cells and comparably low expression on systemic Treg cells (Supplementary Fig. 6). Agonistic engagement of GITR reduces Treg cell numbers in patients with solid tumors, including lung and bladder cancer, although GITR monotherapy failed due to insufficient CTL function^45^. Thus, the infiltration of CTLs in GBM tumors in mice elicited by radiotherapy enabled αGITR-mediated therapeutic response^21^. Moreover, preclinical models that had sufficient numbers of intramural CTLs and were therapeutically responsive to αPD1, were sensitized to αGITR therapy with various treatments^18–21,67^. By contrast, our preclinical models of GBM have limited number of CTLs similar to human GBMs, and thus αPD1 alone did not confer a survival benefit (Fig. 1, Table 1). In addition, αGITR did not reduce intratumoral Treg cells but converted them toward an effector phenotype that expresses Th1-signature genes (Figs. 3–4). In our study, the therapeutic benefit of the αPD1 + αGITR combination is predominantly mediated by this conversion of Treg cells rather than by re-activating CD8 T cells alone (Fig. 5). These findings are in contrast with the data from subcutaneously implanted, non-CNS mouse tumor models, in which the antitumor effect of αPD1 + αGITR therapy is primarily driven by CD8 T cells^67^.

Although acquisition of unstable Treg phenotypes can in general be linked to a reduced FoxP3 expression, there are also studies showing that dedifferentiation of Treg cells can occur while retaining FoxP3 expression^68^. In view of the formation of a multimolecular complex by FoxP3 to condition the transcriptional signature, FoxP3 can activate or repress gene expression in Treg cells depending on its existence in distinct complexes. For example, binding with molecules such as IKZF2 (Helios) and KAT5 renders FoxP3 an activator while its binding to EZH2 and YY1 makes it a repressor^69^. Therefore, it is reasonable to speculate that engagement of GITR that results in alteration of Treg signature genes including Helios may lead to disrupted formation of FoxP3 binding multimolecular complexes, which in turn induces an unstable Treg phenotype. In addition, increased expression of T-bet and Stat4 after αPD1 + αGITR treatment (Fig. 3d) can directly result in epigenetic remodeling of the IFNγ locus leading to activation of IFNγ expression^70^. Molecular changes in Treg cells associated with αPD1 + αGITR treatment, therefore, can lead to dedifferentiation of Treg cells into effector T cells without reduction of FoxP3 expression.

Altogether, the impact of converted Treg cells on enhancing antitumor immunity in GBM highlights two important features of the therapeutic efficacy of this combination: (1) conversion of Treg cells into T effector cells is the major underlying mechanism for the therapeutic effect of αPD1 + αGITR treatment in GBMs; and (2) the Treg cell-dependent efficacy of αPD1 + αGITR appears to be disease context-dependent, in this case likely due to the GBM TME and the potency of Treg cells in regulating the immune suppression in GBM.

IFNγ produced by converted Treg cells and CD8 T cells after αPD1 + αGITR combination therapy may also have an additional effect of driving the reprogramming of subsequent waves of tumor Treg cells, as suggested by observations made in the context of *Helios*^−/−^*, NRP1*^−/−^, and/or *CARMA*^−/−^ Treg cells^40,58,68^. It has been shown that IFNγ produced by *NRP1*^−/−^ Treg cells was able to reprogram wild-type Treg cells, intensifying an antitumor response. While IFNγ produced by CD8 T cells (Fig. 2k) may not be sufficient to drive the initial reprogramming of Treg cells^68^, increased quantity of IFNγ from both reprogrammed Treg cells and CD8 T cells following αPD1 + αGITR treatment (Fig. 2a-l) may elicit additional Treg cell conversion.

Currently, up to two-thirds of patients treated with conventional immune checkpoint inhibitors develop immune-related adverse events^71^, highlighting the need for immunotherapies tailored to the specific immunological milieu in each tumor type. Using three experimental GBM models, our study shows that αGITR treatment preferentially targets tumor Treg cells and sensitizes the tumors to αPD1 therapy. Our finding that converted Treg cells generate a localized memory response restricted to dLNs that does not extend systemically, suggests these converted GBM-specific Treg cells would likely not attack non-CNS organs. Recent studies also demonstrated that Treg cells that are expanded in the brain post-injury are neuroprotective^36^. This may account for why we did not observe any noticeable neurological damage in cured mice. Future studies are needed to fully characterize the long-term impact of these therapies on non-CNS organs.

Collectively, our data (i) provide insights into Treg cell-mediated resistance in experimental GBMs, (ii) demonstrate that reprogramming the GBM TME may differ significantly from peripheral tumors, (iii) offer a possible solution to overcome GBM resistance to immunotherapy, and (iv) provide mechanistic insight into how alleviating Treg cell-mediated resistance combined with a reinvigoration of CTLs cooperates in exerting an antitumor effect. Given that αGITR antibodies are currently being evaluated in a number of clinical trials (NCT04021043, NCT02740270, NCT02628574, NCT02598960, and NCT04225039), our study provides mechanistic insights and compelling preclinical data in support of testing the combination of αGITR with αPD1/PDL1—with or without the SoC therapy—in GBM patients with a high intratumoral accumulation of Treg cells.

## Methods

### Antibodies

For ease of identification, full details of antibodies are shown under the registry of publicly available data base (antibodyregistry.org) that provides a comprehensive list of suppliers, and appropriate references for usage. This information is summarized under “antibody registry number” in the Supplementary Table 1 included in the supplementary information file.

### Mice

C57BL/6, *FoxP3*^*DTR*^, and *Rag1*^−/−^ mice were obtained from the Cox-7 gnotobiotic animal facility operated by the Edwin L. Steele Laboratories, Department of Radiation Oncology at the MGH. All animal experiments were performed in the Cox-7 animal facility, accredited by the Association for Assessment and Accreditation of Laboratory Animal Care International. Female and male mice were used. Animal protocols were approved by the Institutional Animal Care and Use Committees at MGH.

### Cell culture

The murine GL261 GBM cell line (GL261-WT) was provided by the Frederick National Laboratory (National Cancer Institute, NCI) and was grown in serum-free conditions using the NeuroCult NS-A proliferation kit (Stemcell Technologies). The murine CT2A was obtained from Dr. Thomas N. Seyfried’s laboratory at Boston College^72^ and cultured in Dulbecco’s Modified Eagle Medium supplemented with 10% fetal bovine serum and 1% penicillin-streptomycin (10,000 U/mL). 005GSC cells were obtained from Dr. Samuel D. Rabkin laboratory^73,74^. 005GSC resistant clones to αPD1 was further selected in mice (three rounds of in vivo selection) to ensure both stem-like features and resistance to immunotherapy. Myc-MB cell line was a generous gift from Dr. Martine F. Roussel at St. Jude Children’s Research Hospital^75^. MB tumor-spheres were cultured in Neurobasal medium supplemented with N2, B27, human recombinant basic fibroblast growth factor, and epidermal growth factor (EGF) (PeproTech) in ultra-low attachment flasks. The murine 4T1 breast cancer cell line was obtained from ATCC and maintained in DME media supplemented with 10% FBS and 1% Penicillin /Streptomycin. Murine breast tumor cells that were selected for their metastasis to the brain [ERB (ErbB2-P) cells] were received from the Massagué lab at Memorial Sloan Kettering Institute (NY)^76^, and cultured in 10% FBS and 1% Penicillin /Streptomycin. The GL261-MGH and CT2A cell lines expressing secreted Gaussia luciferase (GLUC) were generated by transducing cells with a lentiviral vector co-expressing GLUC and GFP^63^, provided by the MGH vector core, followed by sorting. The 005GSC cell line expressing secreted GLUC was generated by transfecting cells with a GLUC plasmid followed by puromycin selection. All cell lines were grown in a humidified atmosphere of 5% CO2 and 95% air at 37 °C and repeatedly tested and were negative for mycoplasma using the Mycoalert Plus Mycoplasma Detection Kit (Lonza).

### Flow cytometry

Single-cell suspensions were prepared from each organ (tumor, LN, spleen, and blood). Specifically, tumor-only regions were isolated under the stereotactic microscope, followed by single-cell preparation. Cells were stained with antibodies listed in the core resource table with concentrations of 1:200 for all targets except Helios that was stained at a concentration of 1:50 and CD44 at a concentration of 1:400. Surface staining was performed on ice for 20 min. For cytokine expression analysis, cells were activated with Leukocyte Activation Cocktail (BD Biosciences, Cat no: 554656) in RPMI containing 10% FBS and 1% penicillin-streptomycin (10,000 U/mL) for 4.5 h. For the flowcytometric analyses, cells were first stained with surface markers in FACS buffer (2% BSA in PBS) for 20 min on ice. Then cells were fixed in Fix/Perm buffer (eBioscience) for 15 min, washed in permeabilization buffer (eBioscience), and stained for intracellular factors in permeabilization buffer for 20 min on ice. Live cells for transfer experiments and in vitro assays were sorted using BD FACSAria^™^ II cell sorter (BD Biosciences). Cells were analyzed on Aria II (BD Biosciences) or LSRII (BD Biosciences), and data analysis was performed on FlowJo (Tree Star 10.7v).

### Tumor models

C57BL/6, *Foxp3*^*DTR*^*-GFP*, or *Rag1*^−/−^ mice were injected with tumor cells (1 × 10^5^ GL261-MGH-GFP-GLUC, 5 × 10^4^ CT2A-GFP-GLUC, 7.5 × 10^4^ 005GSC-GFP-GLUC, 1 × 10^5^ Myc-MB, 5 × 10^3^ 4T1, 5 × 10^4^ ERB) orthotopically using a stereotactic device. All brain tumor cells were implanted in the forebrain except for Myc-MB cells that were implanted in the cerebellum. Tumor size was measured either by micro-ultrasound, blood GLUC, or both^7^. Tumors were size-matched and randomized to treatment groups. After treatment initiation, tumor size was monitored every 3 days by blood GLUC measurements.

For survival studies, C57BL/6 mice bearing GL261-MGH-GFP-GLUC, CT2A-GFP-GLUC, or 005GSC-GFP-GLUC were treated every 3 days with total six doses of (i) IgG2a; (ii) αPD1 (Bioxcell, RMP1-14); (iii) αGITR (Bioxcell, DTA-1); or (iv) αPD1 + αGITR. Tumor size was measured every 3 days until the first mice showed clinical signs of GBM associated morbidity (including serious movement problems, hunch-back, and/or weight loss beyond 15%), at which time the mice were euthanized.

*Rag1*^−/−^ mice received (i) vehicle, (ii) 5 × 10^5^ CD4 Treg cells (TCR^+^CD4^+^ CD25^+^CD127^lo^), (iii) 1 × 10^6^ CD8 cells (TCR^+^CD8^+^), or (iv) 5 × 10^5^ CD4 Treg cells and 1 × 10^6^ CD8 T cells. Mice were inoculated with GL261-MGH-GFP-GLUC or CT2A-GFP-GLUC 2 days after cell transfer. For each set of cell transfer experiments, mice were randomized to two cohorts post tumor establishment (size 0.5–1 mm^3^) for treatment with IgG2a or αPD1 + αGITR (*n* = 4–6 per cohorts). They were then treated with six doses of each antibody (250 µg/mouse/dose). Tumor size based on blood GLUC was measured until the first mouse showed clinical signs of the GBM associated morbidity, as stated above.

For immunological assays, mice were sacrificed 2–3 days after the 4th treatment. Tumor, LNs, blood, and spleen were harvested. Single-cell preparation (including TILs) was conducted using a modified mechanical dissociation method^8^ and cells were analyzed as previously described in the antibody and flow cytometry section.

For multi-arm treatment studies, 8–12 mice were assigned to each group, to achieve statistical power. For two-arm cell transfer experiments, at least 4–5 mice were used in each group. Tumor growth was analyzed using two-way ANOVA with multiple comparisons. Event-free survival (moribund) estimates were calculated with the Kaplan–Meier method. Groups of mice were compared by log-rank test.

For the SoC study, cranial window bearing^77^ mice were implanted with CT2A-GFP-GLUC cells. When tumors reached sizes between 5 and 10 mm^3^, as measured by their GLUC value, tumors were surgically excised, and mice were kept under observation for 3 days. Then mice were assigned to treatment groups and treated with 10 days of consecutive radiation (1G per day) and chemotherapy TMZ (25 mg/kg, i.p.).

### Human samples

This study was approved by the Institutional Review Board at Brigham and Women’s Hospital (BWH). Human Glioblastoma samples were identified through the surgical pathology files at the BWH. All of the patient samples were de-identified prior to the study. Patient samples were collected as part of SoC treatment. No informed consent was obtained since this was a retrospective study in which excess tissue was used from otherwise consented procedures as part of routine clinical care. Patient records were reviewed for age, sex, medical history, surgical intervention, therapeutic regimens, IDH1 mutation status, MGMT promotor methylation, cytogenetics and overall survival. All patients (except 1 patient which opted for no treatment) received SoC therapy ± immune checkpoint blockade ± Bevacizumab.

PBMCs were collected from healthy volunteers and not cancer patients. The data were anonymized and categorized under MGH general informed consent protocol.

### RNA-seq analysis of Treg cells

Treg cells were sorted from tumor tissues or spleens of treated mice. Next-generation sequencing libraries were prepared according to the Smart-Seq2 protocol as previously described^78–80^.

Briefly, RNA from cell lysates were captured using Agencourt RNA Clean beads then converted to cDNA. The whole transcriptome was amplified before quantification and dual-index barcoding with an Illumina Nextera XT kit. The cDNA libraries were enriched then pooled in equal volumes before final bead clean-up and sequencing on a NextSeq500 instrument. Reads were aligned to mm10 mouse reference genome using Tophat, and RSEM^81^ was used to calculate transcripts per million. Normalized read counts were filtered for robust expression (>10) and low CV and processed in the Multiplot suite and Morpheus (https://software.broadinstitute.org/morpheus/).

### In vitro co-culture assays

In preclinical models, Treg cells or CD8 T cells were isolated from tumors, spleen, or LN of tumor-bearing mice or naive C57BL/6 mice. Cell sorting was done post enrichment using Treg cell isolation or CD8 isolation kits. Pre-sorting enrichment assays in mice are done using commercial kits. Treg cells were enriched using CD4 + CD25+ Regulatory T Cell Isolation Kit (Miltenyl biotech, 130-091-041) and CD8 with Naive CD8a+ T Cell Isolation Kit, mouse (Miltenyl biotech, 130-096-543). Culture media was Roswell Park Memorial Institute medium (RPMI) supplemented with 10% FBS and 1% penicillin-streptomycin. Two models of co-culture assay were performed. In one setting, Treg cells and CD8 T cells (1:1 ratio) were co-cultured with or without tumor cells in the presence of αCD3/CD28 beads (Biolegend), and their phenotype and cytokine production were measured using flow cytometry within the first 3 days. For experiments with long term culture (>3 days), IL-2 (20 ng/mL) was added to the culture. In another setting, tumor cells (1 × 10^3^ cells) were cultured overnight in 96 round bottom plates with 100 µL of their growth media. After 24 h, sorted Treg cells or CD8 T cells were added to tumor cells in the ratios of 1:1, 1:2, 1:5, or 1:10, and tumor cell viability was measured by MTT assay at 48 h. The proliferation of tumor cells was assessed by GLUC at 24 and 48 h. Co-culture assay for neutralization experiment was performed in the presence of 10 µg/mL αIFNγ (Biolegend), αMHC Class I (BioXcell, Y3), αMHC Class II (BioXcell, M5/114), αTNFα (Biolegend), αGzmB, and αFAS L (Biolegend, MFL4) antibodies.

For human co-culture studies, Treg cells and CD8 T cells were FACS sorted from already prepared PBMC (Mark Cobbold laboratory at MGH cancer center). CD8 and Treg cells were before in vitro assays. For T cell expansion, human IL-2 (20 ng/mL) and dynabeads human T-Activator CD3/CD28 (Thermo fisher) were added to the culture media. Then, Treg cells or CD8 T cells were added to human GBM tumor cells (MGG4-GFP-GLUC and MGG8-GFP-GLUC) under therapy conditions and blockade as stated above, and tumor cell proliferation was measured.

### Quantification and statistical analysis

Statistics were performed using Prism v6.07 and v8. One-way analysis of variance (ANOVA) with Tukey’s post hoc test was used as indicated in the figure legends. The Kaplan–Meier method was used for survival studies as indicated in the figure legends. Student’s *t* test was used for two-arm studies as indicated in the figure legends. N represents the number of mice used in the experiment, with the number of individual experiments listed in the legend. Graphs show individual or in case of survival studies combined experiments/samples. Results are presented as mean with or without error bars showing the standard error of the mean (SEM). Differences with *p* < 0.05 were considered statistically significant.

### Reporting summary

Further information on research design is available in the Nature Research Reporting Summary linked to this article.

## Supplementary information

Supplementary Information

Reporting Summary

## Data Availability

All data generated by this study are available within the Article, Supplementary Information or available from the authors upon request. Source data are provided with this paper. Our RNA sequencing data from GBM Tregs vs. spleen Tregs are available in Gene Expression Omnibus (GEO) under accession code GSE167746. Materials are available under a material transfer agreement (contact person R.K.J.). Source data are provided with this paper.

## Source data

Source Data
