# Supplementary material for: Targeting Treg cells with GITR activation alleviates resistance to immunotherapy in murine glioblastomas
Source: Nat Commun. 2021 May 11;12:2582. doi: 10.1038/s41467-021-22885-8 (PMC8113440; doi:10.1038/s41467-021-22885-8)
Supplement: Supplementary file 1 — Supplementary Information [file 41467_2021_22885_MOESM1_ESM.pdf]

Supplementary Information file

Targeting Treg cells with GITR activation alleviates resistance to immunotherapy in murine glioblastomas

Zohreh Amoozgar[1,†], Jonas Kloepper[1,†], Jun Ren[1,†], Rong En Tay[2], Samuel W. Kazer [3], Evgeny Kiner[4], Shanmugarajan Krishnan[1], Jessica M. Posada[1], Mitrajit Ghosh[1], Emilie Mamessier[1], Christina Wong[1], Gino B. Ferraro[1], Ana Batista[1], Nancy Wang[1], Mark Badeaux[1], Sylvie Roberge[1], Lei Xu[1], Peigen Huang[1], Alex K. Shalek[3], Dai Fukumura[1], Hye-Jung Kim[2,*], Rakesh K. Jain[1,*]

[†] These authors have contributed equally
* Correspondence to: Rakesh K. Jain (Jain@steele.mgh.harvard.edu) and Hye-Jung Kim (Hye-Jung_kim@dfci.harvard.edu)

Supplementary Table 1: Antibodies List.

For ease of identification, full detail of antibodies and materials are shown under the registry of publicly available data base (antibodyregistry.org) that provides a comprehensive list of suppliers, and appropriate references for usage. These information is summarized under antibody registry number in the listed table.

Reagent (antibodies)	Concentrations	Provider	Clone and antibody registry number
CD4	1/200	BD Pharmingen	RM4-5; RRID: AB_1937327
CD4	1/200	BDHorizon	GK1.5; RRID:AB_395010
CD8	1/200	Biolegend	53-6.7; RRID: AB_2563056
CD11b	1/200	eBioscience	M1/70; RRID:AB_468883
CD11c	1/200	Biolegend	N418; RRID:AB_313770
CD11c	1/200	BD Biosciences	HL3; RRID:AB_395058
CD206	1/200	Biolegend	C068C2; RRID: AB_2562232
CD39	1/200	Biolegend	Duha59; RRID:AB_2563394
CD45	1/200	Biolegend	30-F11; RRID:AB_10898325
CD49b	1/200	Biolegend	HMa2; AB_1595599
CD62L	1/200	eBioscience	MEL-14; AB_469632
CD69	1/200	Biolegend	H1.2F3; RRID:AB_10683447
CD73	1/200	Biolegend	TY/11.8; RRID AB_2154094
CD127	1/200	BD Pharmingen	SB/199; RRID: AB_469649
CD127	1/200	eBioscience	A7R34; AB_469435
CTLA4	1/100	eBioscience	UC10-4B9; RRID:AB_467510
F4/80	1/200	Biolegend	BM8; RRID: AB_893486
FoxP3	1/100	Biolegend	150D; RRID: AB_439747
FoxP3	1/100	Biolegend	FJK-16s; RRID: AB_1518812
FR4	1/200	eBioscience	eBio12A5; RRID: AB_842799
GITR	1/200	BD Horizon	DTA-1
GITR	1/200	Biolegend	YGITR765; RRID: AB_528907
GR1	1/200	eBioscience	RB6-8C5
GR1	1/200	BD Horizon	RB6-8C5
GzmB	1/200	eBioscience	GB11; AB_2114575
Helios	1/50	Biolegend	22F6; RRID: AB_10662900
IL-10	1/100	Biolegend	JEs5-16E3; RRID: AB_11150582
IL-2	1/100	Biolegend	JES6-5H4; RRID: AB_2650897
INFg	1/200	Biolegend	XMG1.2; RRID: AB_493312

KLRG1	1/200	BD Horizon	2F1
Lag3	1/200	Biolegend	C9B7W; RRID: AB_2561516
OX-40	1/200	Biolegend	OX-86; RRID: AB_10962569
PD1	1/200	Biolegend	29F.1A12; RRID: AB_2159184
PD1	1/200	Biolegend	29F.1A12; RRID: AB_2563680
TCRb	1/200	Biolegend	H57-597; RRID: AB_2562350
TIGIT	1/200	Biolegend	1G9; RRID: AB_10962572
Tim3	1/200	eBioscience	8B.2C12; AB_2573233
TGFβ	1/100	Biolegend	TW7-16B4; AB_2561580
TNFα	1/200	Biolegend	MP6-XT22; AB_2565953
αMHC Class I (H-2K^b)	1/200	Biolegend	28-8-6; AB_313598
αMHC Class II	1/200	Biolegend	M5/114.15.2; AB_313328

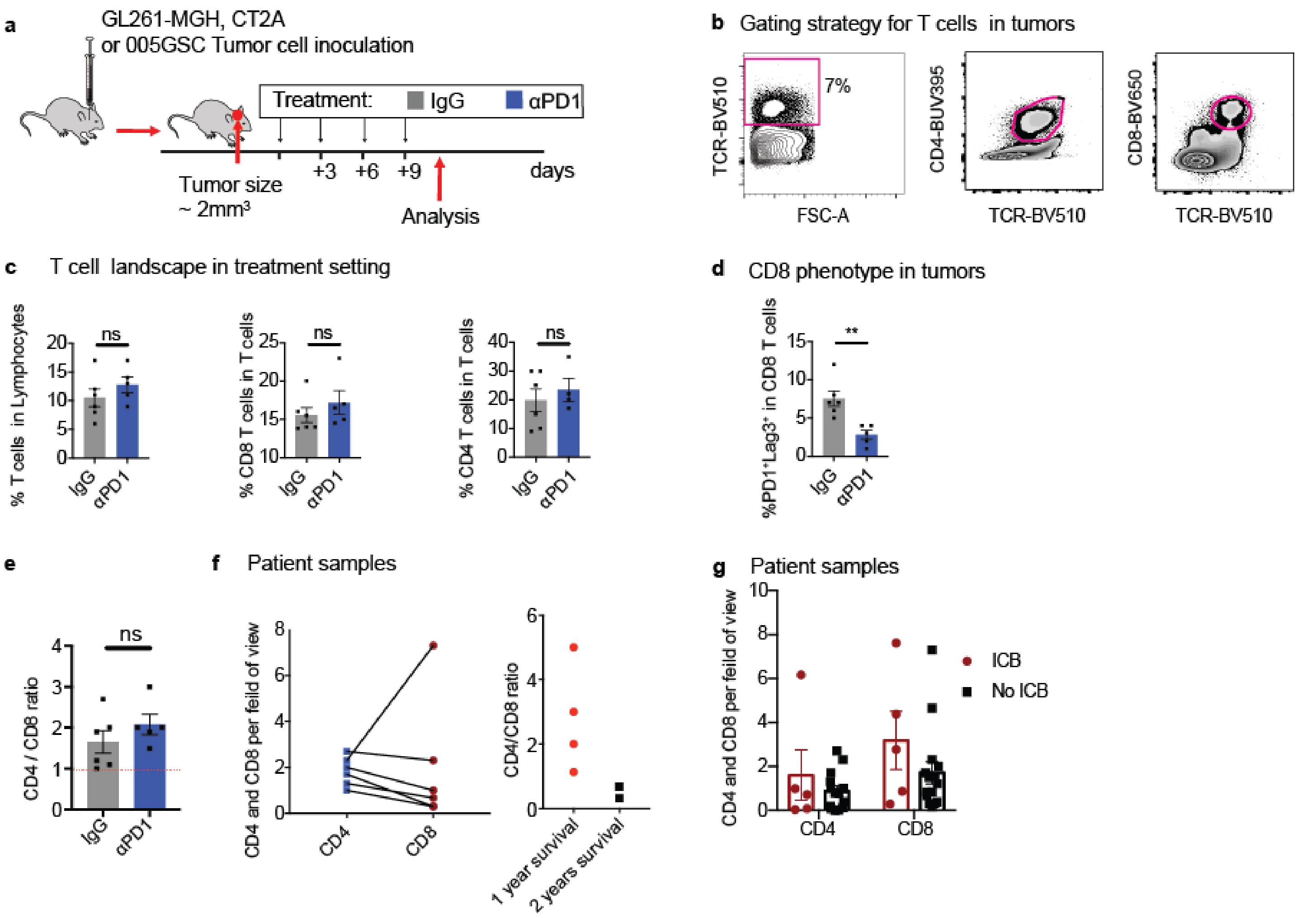

Supplementary Figure 1. T cell landscape in brain tumors with and without ICB therapy

a) Schematic representation of ICB treatment protocol during GBM tumor challenge. Mice were inoculated orthotopically with $1x10^5$ GL261-MGH-GFP-GLUC cells. Mice were randomized into two groups (tumor size of ~2 mm^3 as measured by blood GLUC activity (n=11)) and then treated with 4 doses of i) IgG2a (isotype control) (n=6), and ii) αPD1 (n=5) administered every 3 days intraperitoneally. The initial treatment dose was 500 µg/dose/mouse, followed by 3 maintenance doses of 250µg for both antibodies. Two independent repeats of experiments.

b) Gating strategy for T cells in brain tumors.

c) T cell frequencies within brain tumors after the last treatment, as stated in (a) [(IgG (n=6) and αPD1 (n=5) biological replicates)]. IgG2a and αPD1 therapies were compared with Two-tailed unpaired t-test [(% T cells, p=0.306, not significant), (% CD8 in T cells, P=0.3673, not significant), and (% CD4 in T cells, P=0.5491, not significant)]. Data presented are mean ± SEM.

d) Frequency of PD1 and Lag3 double-positive cells in intratumoral CD8 T cells in GBM tumors treated as in (**a**). IgG2a and αPD1 therapies were compared with Two-tailed unpaired t-test, p= 0.0038, **p < 0.01. Data presented are mean ± SEM.

e) The ratio of CD4 to CD8 T cells in tumors after each treatment (see a**)**. P=0.2805, not significant. Data presented are mean ± SEM.

f) Number of CD4 and CD8 T cells in clinical samples from initial diagnosis or at autopsy (n=6). All patients had received the standard of care (SoC), including resection of GBM tumors, chemotherapy (temozolomide) and radiation therapy [1].

g) Quantity of CD4 and CD8 T cells in clinical samples from GBM patients (n=20) that underwent SoC with or without ICB treatments. Data presented are mean ± SEM.
Source data are provided in a Source Data file.

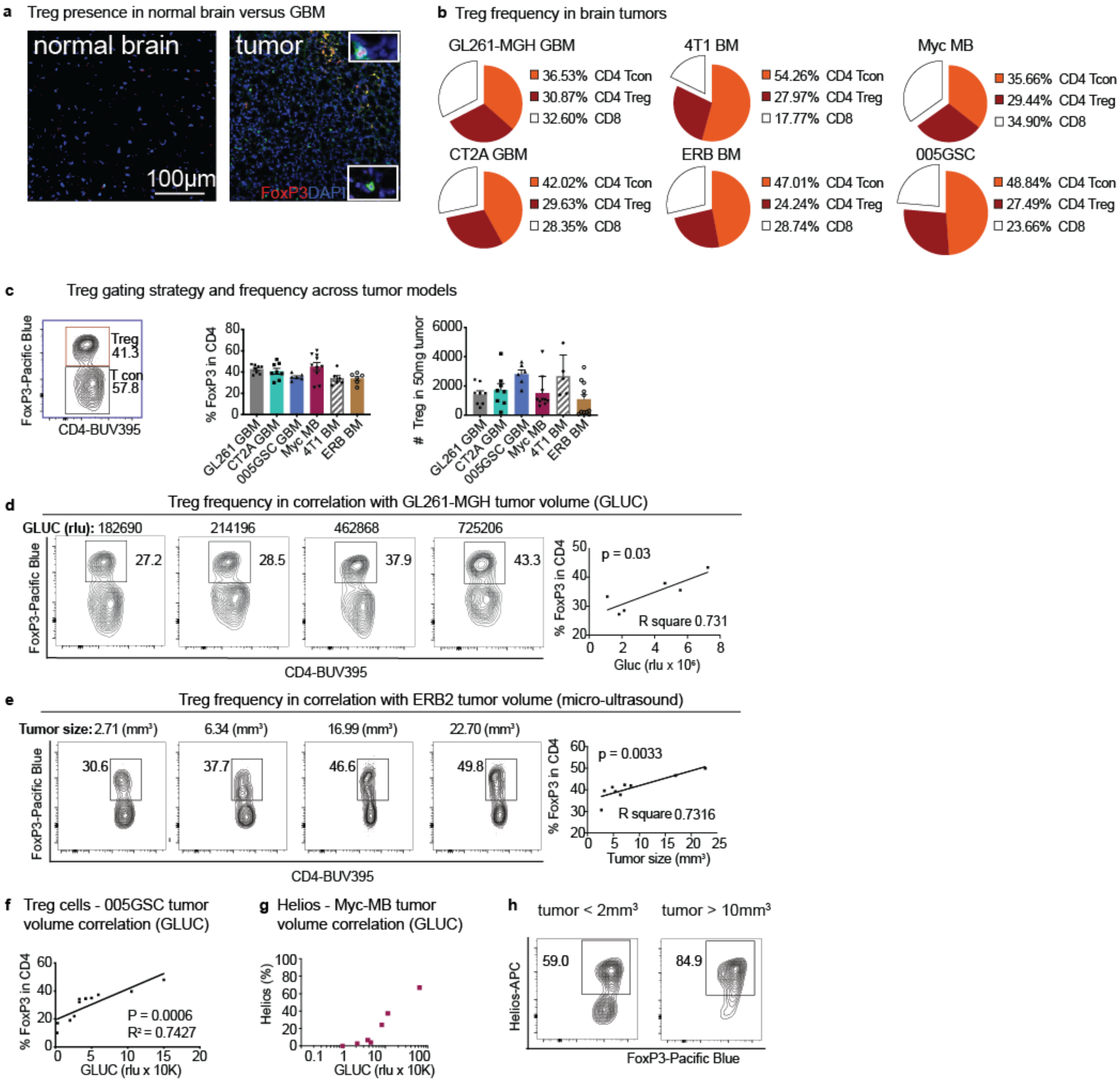

Supplementary Figure 2. Treg cell frequency and anergy increase proportionally to tumor size.

a) Representative micrographs showing CD4 FoxP3$^+$ T cells (CD4 Treg cells) in healthy mouse brain (left) and in GL261-MGH GBM tumors grown orthotopically in mice (right). Two independent repeats of experiments.

b) Profile of T cells in primary adult GBM tumors (GL261-MGH, CT2A, and 005GSC), metastatic pediatric tumors (group 3 Myc medulloblastoma), and breast cancer brain metastases (4T1 and ErbB2).

c) Treg cell gating strategy and frequency and number within T cells in primary adult GBM tumors [(GL261-MGH (n=8), CT2A (n=8), and 005GSC (n=6)], metastatic pediatric tumors [(group 3 Myc medulloblastoma) (n=10)], and breast cancer brain metastases [(4T1 (n=6) and ErbB2 (n=6)]. Data presented are mean ± SEM. p= 0.021 for Treg cell frequency and p=0.5305 for Treg cell number, by one-way ANOVA test and corrected for multiple comparisons using the Tukey adjustment. Between group comparison in Treg cell frequency did not show statistical differences. Each n is a biological replicate.

d) Correlation between the frequency of tumor-infiltrating Treg cells and the size of orthotopic GL261-MGH tumors (determined by GLUC values) (n=6), using linear regression test.
e) Correlation between the frequency of tumor-infiltrating Treg cells and the size of breast cancer metastasized to the brain (ERB2 tumors measured by ultrasound) (n=9), using linear regression test.
f) Correlation between the frequency of tumor-infiltrating Treg cells and the size of orthotopic G005GSC tumors (determined by GLUC values) (n=11), using linear regression test.
g) Correlation between the fraction of Helios expressing intratumor Treg cells and Myc-MB tumor volume (n=7), using linear regression.
h) Helios expression in Treg cells in representative small (~2 mm^3) and large (>10 mm^3) GL261-MGH tumors.
p-values in panels **d** and **e** were calculated using a linear regression test. $*p < 0.05$, $**p < 0.01$, $***p < 0.001$.
Source data are provided in a Source Data file.

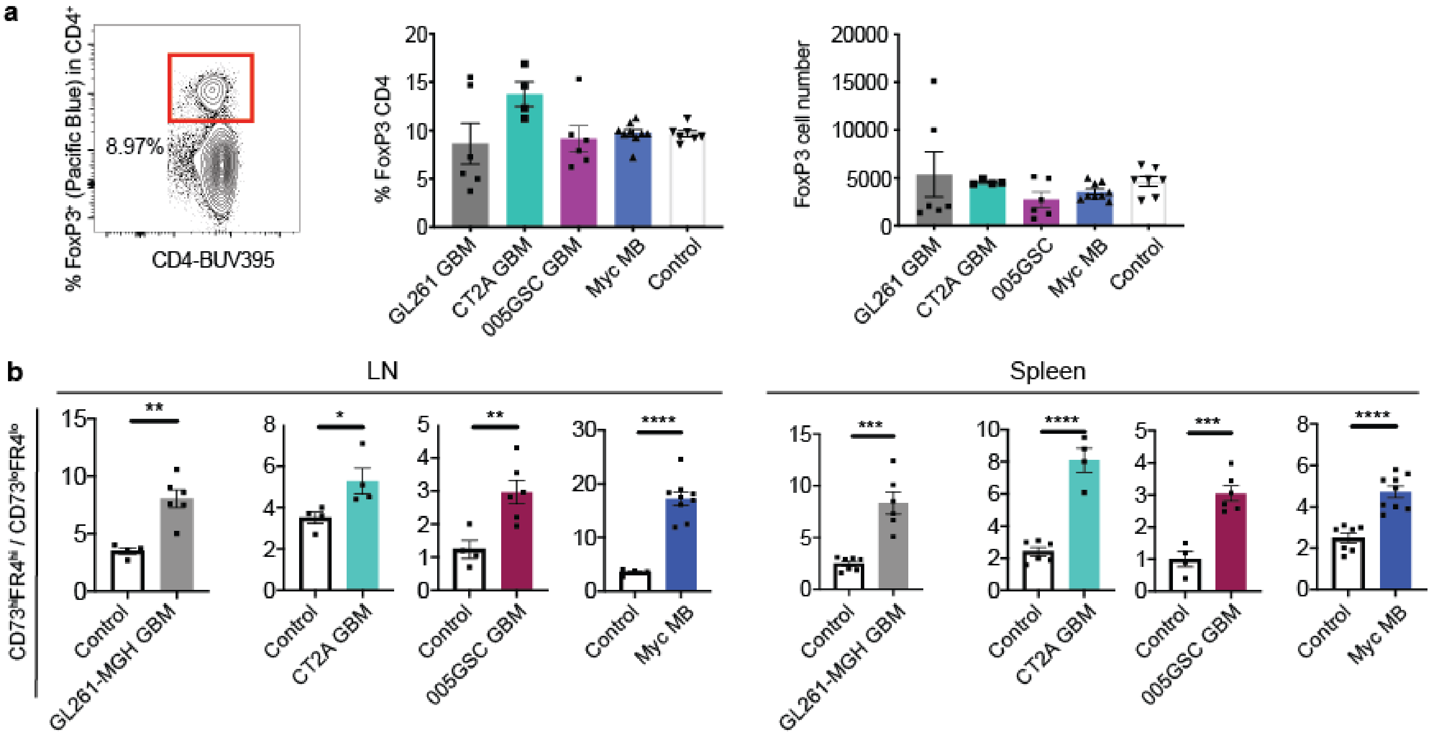

Supplementary Figure 3. Treg cell anergy increases in peripheral lymphoid organs of brain tumor-bearing animals.

a) Treg cell gating strategy (left) and cell numbers in spleens and cervical LNs of tumor-free [control (n=7)] or tumor-bearing mice: GBM [(GL261-MGH (n=6), CT2A (n=4) , or 005GSC (n=6)] or metastatic medulloblastoma (Myc-MB, n=9). N represents biological replicates. Data presented are mean ± SEM. p= 0.0892 for Treg cell frequency and p=0.4978 for Treg cell number, by one-way ANOVA test. Shown are combined data from two independent repeats of experiments.

b) The ratio of Treg cells co-expressing high and low levels of CD73 and FR4 in LN and spleen. Data presented are mean ± SEM. Control group was compared with tumor bearing mice using Two-tailed unpaired t-test. LN: GL261-MGH p=0.0017, 95% confidence interval 2.289 to 6.811; CT2A p=0.0416, 95% confidence interval 0.09340 to 3.457; 005GSC p=0.0081, 95% confidence interval 0.5885 to 2.866; Myc-MB p<0.0001, confidence interval 9.498 to 17.95. Spleen: GL261-MGH p=0.0001, 95% confidence interval 3.642 to 8.086; CT2A, p<0.0001 95% confidence interval 4.139 to 7.227 ; 005GSC p=0.0004, 95% confidence interval 1.240 to 2.855; Myc-MB p<0.0001, confidence interval 1.428 to 3.045. *p < 0.05, **p < 0.01, ***p < 0.001, **** p < 0.0001. Source data are provided in a Source Data file.

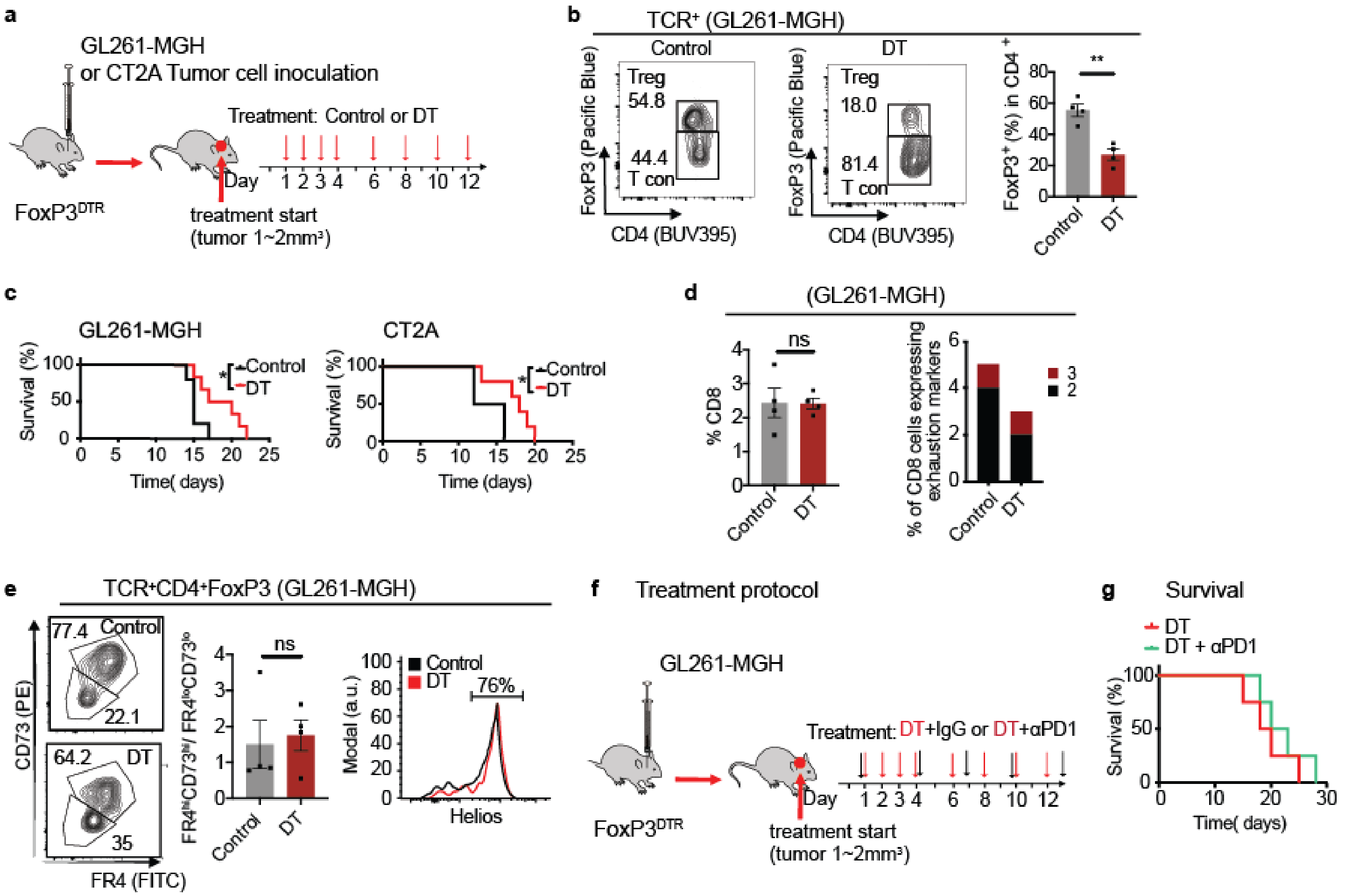

Supplementary Figure 4. Low numbers of Treg cells are sufficient to suppress CD8 T cell function and resist αPD1 treatment.

a) Schematic representation of experimental protocol to evaluate the effect of Treg cell depletion on GBM tumor growth. Gaussia luciferase (GLUC) expressing GL261-MGH (1X10^5 cells) or CT2A (5X10^4 cells) tumor cells were orthotopically inoculated in *FoxP3^{DTR}* mice. The experimental animals were randomized by GLUC values corresponding to tumors 0.5 - 1.5 mm^3 in volume. Mice were given PBS control (n=5) or diphtheria toxin (DT; n=5) intraperitoneally every day for 4 days and every other day for an additional 4 (for CT2A tumors) or 6 (for GL261-MGH tumors) doses. N represent biological replicates of two independent repeats of experiments.

b) Representative flow cytometry plots (left) and quantification (right) of Treg cell depletion after DT treatment. Data presented are mean ± SEM. Control (n=4), DT (n=4). p=0.0017 by Two-tailed unpaired t-test (95% confidence interval -41.57 to -15.53). N represents biological replicates of two independent repeats of experiments.

c) Survival of mice challenged with GL261-MGH and CT2A tumors and treated as in (**a**). Survival was assessed using Kaplan-Meier survival analysis. *p < 0.05. N represent biological replicates of two independent repeats of experiments. Number of mice is each group and Median survival: [GL261-MGH Control (n=5, 15 days), DT (n=5, 18.5 days)] and [CT2A Control (n=4, 14 days), DT (n=4, 18 days)].

d) *Left*, Frequency of CD8 T cells in live cells in GL261-MGH tumors (n=4 control and DT treated). p=0.9707, statistically insignificant. *Right*, Fraction of CD8 T cells expressing 2 or 3 markers of the suppressive function (PD1, Lag3, TIGIT) in GL261-MGH tumors treated as in (**a**). N represent biological replicates of two independent repeats of experiments. Data presented are mean ± SEM.

e) Representative flow cytometry plots of co-expression of CD73 and FR4 in CD4 Treg cells (left) and quantification of the ratio (right) of $CD73^{hi}FR4^{hi}$ to $CD73^{lo}FR4^{lo}$ CD4 Treg cells within GL261-MGH tumors

treated as in (**a**). p=0.7669, statistically insignificant. Helios expression by CD4 Treg cells with or without induction of Treg cell depletion. N represents biological replicates of two independent repeats of experiments. Data presented are mean ± SEM.

f) Schematic representation of experimental protocol to evaluate whether CD4 Treg cell depletion alone was sufficient to alleviate GBM resistance to ICB. Mice bearing GL261-MGH tumors (~1-2 mm^3 in volume) were treated with 4 doses of DT, and received either anti-PD1 (n=6) or control IgG2a (n=6) in addition to continuing the DT treatment. Median survival: [GL261-MGH Control (19 days), DT (21 days)].

g) Survival of mice treated as in (**f**). Two independent repeats of experiments.

Survival was assessed using Kaplan-Meier survival analysis.

Source data are provided in a Source Data file.

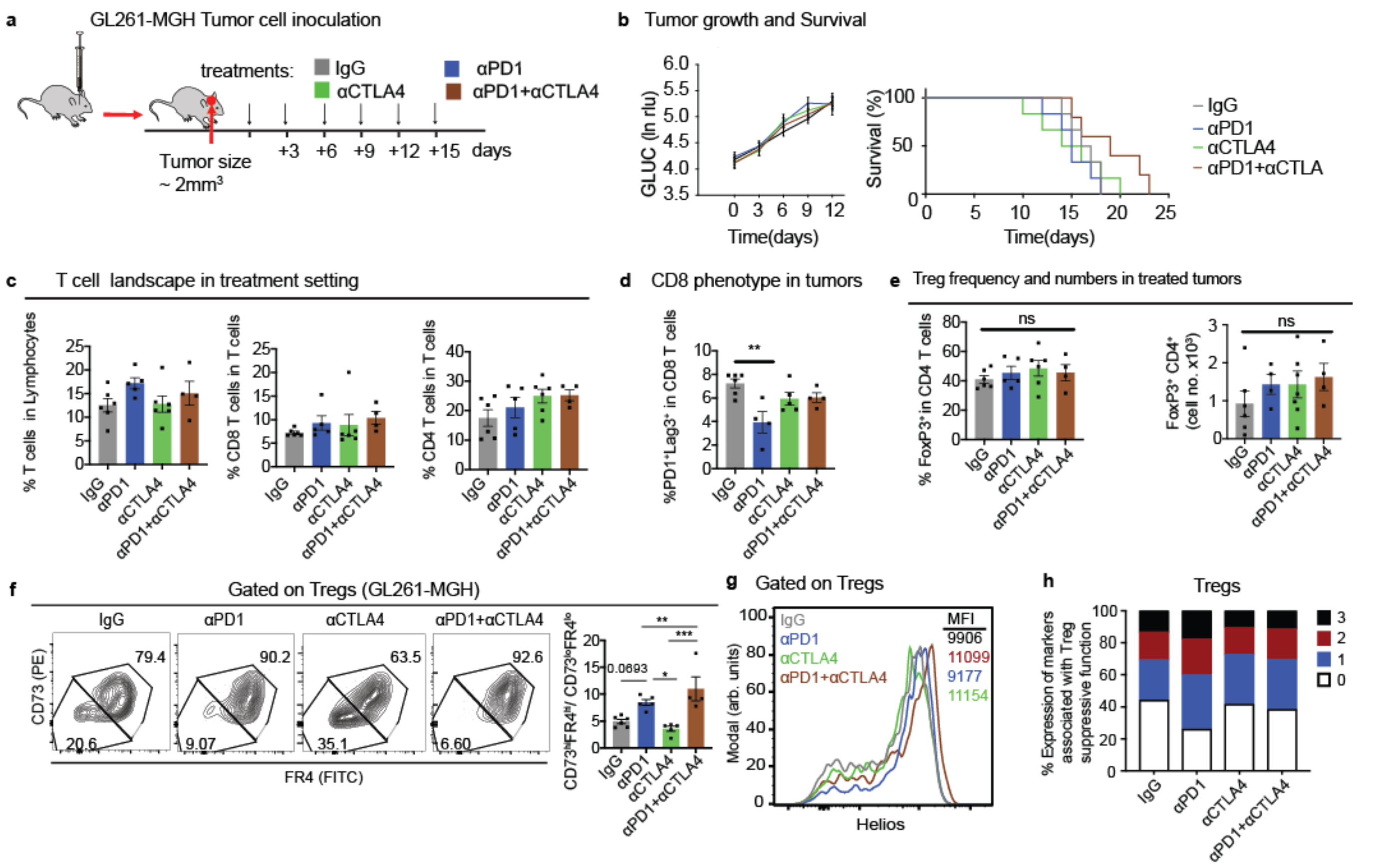

Supplementary Figure 5. GBMs harbor significant numbers of Treg cells with a strongly suppressive phenotype that causes resistance to ICB.

a) Schematic representation of ICB treatment protocol during GBM tumor challenge. Mice were inoculated with 1x10^5 GL261-MGH cells orthotopically and tumor growth was monitored using serial measurements of Gaussian luciferase activity (GLUC) in the blood. Mice were randomized to 4 treatment groups when tumor size reached ~2 mm^3 and then treated with 4 doses of i) IgG2a control (n=7), ii) αPD1 (n=7), iii) αCTLA4 (n=6) or iv) αPD1+αCTLA4 (n=6) administered every 3 days intraperitoneally. Treatment dose was 500µg/mouse, followed by maintenance doses of 250 µg for all antibodies. Data shown are representative of two independent repeats of experiments.

b) Tumor growth and survival of mice treated as in (**a**) (n=6). Tumor growth was compared by two-way ANOVA (Days × Group) (not significant). Survival was assessed using Kaplan-Meier survival analysis (not significant). Data shown are representative of two independent repeats of experiments.

c) T cell frequencies within brain tumors treated as in (**a**), IgG2a control (n=6), αPD1 (n=5), αCTLA4 (n=6) or αPD1+αCTLA4 (n=4). Data shown are mean ± SEM; p=0.2050 by ordinary one-way ANOVA test, insignificant. N represents biological replicates.

d) Expression of PD1 and Lag3 in intratumoral CD8 T cells in GBM tumors treated as in (**a**), IgG2a control (n=6), αPD1 (n=4), αCTLA4 (n=5) or αPD1+αCTLA4 (n=4), analyzed on day 12..Data presented are mean ± SEM P=0.0068 by ordinary one-way ANOVA test, and were corrected for multiple comparisons using the Tukey adjustment. ** refers to p < 0.01. N represents biological replicates.

e) Treg cell frequency and cell number in tumors treated as in (**a**), IgG2a control (n=6), αPD1 (n=5), αCTLA4 (n=6) or αPD1+αCTLA4 (n=4), analyzed on day 12. Data presented are mean ± SEM, p=0.6681 (frequency) and 0.7983 (cell number) by one-way ANOVA test (not significant). N represents biological replicates.

f) Representative flow cytometry plots (left) showing co-expression of CD73 and FR4 in GBM tumor-infiltrating CD4 Treg cells, and quantification of the ratio of $CD73^{hi}FR4^{hi}$ to $CD73^{lo}FR4^{lo}$ CD4 Treg cells within GBM tumors under ICB treatment, IgG2a control (n=6), αPD1 (n=5), αCTLA4 (n=6) or αPD1+αCTLA4 (n=4). N

represents biological replicates. Data presented are mean ± SEM. P=0.0004 by one-way ANOVA test, and corrected for multiple comparisons using the Tukey adjustment. Significance shown as: *p < 0.05, **p < 0.01, ***p < 0.001.

g) Helios expression and Mean Fluorescence Intensity (MFI) of Helios in CD4 Treg cells quantified by FACS.

h) Distributions of expression of markers associated with Treg cell suppressive function (PD1, Lag3, TIGIT) in GBM Treg cells quantified by FACS.

Source data are provided as a Source Data file.

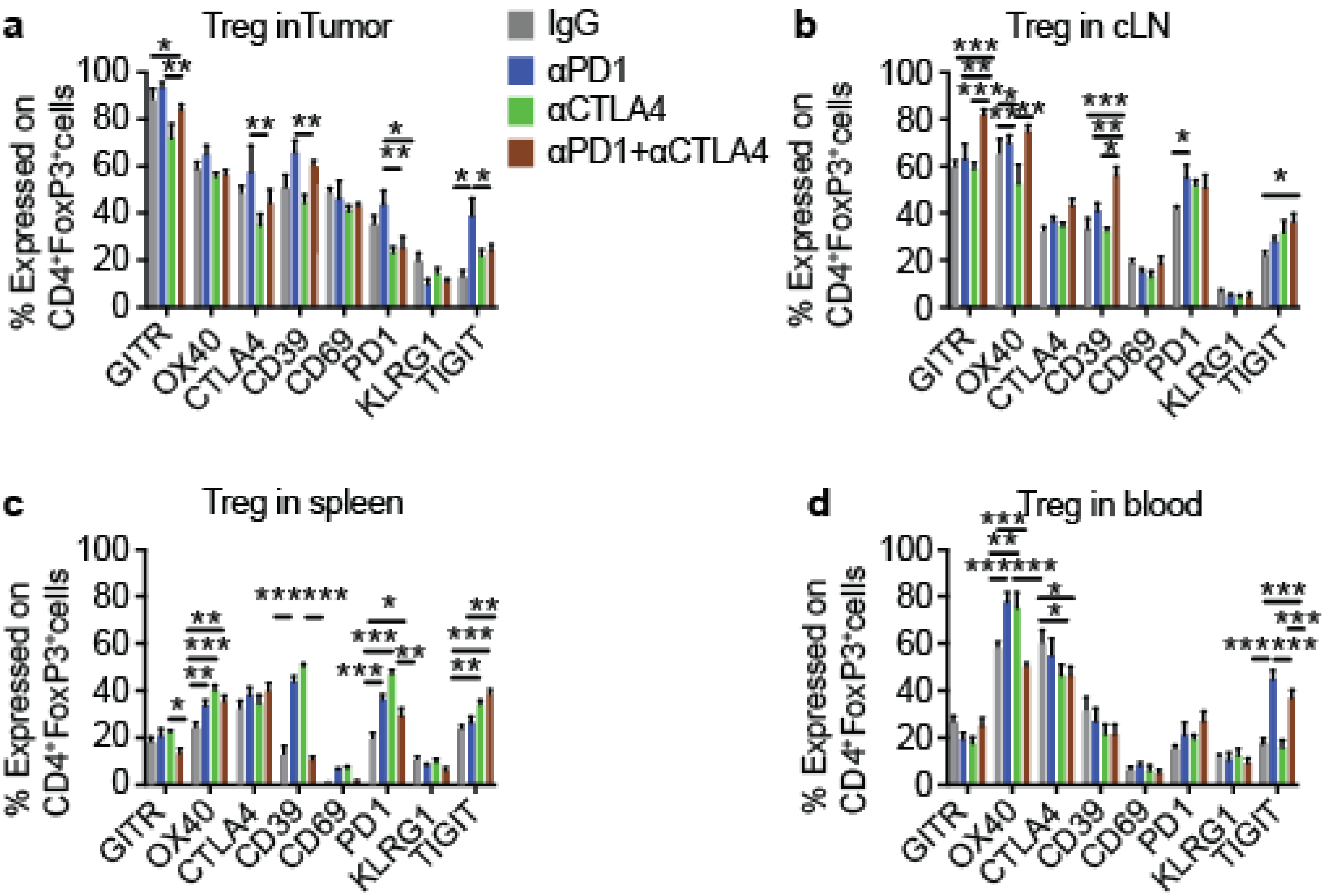

Supplementary Figure 6. GITR is predominantly expressed on Treg cells in the brain TME and is unchanged by ICB therapy.

Treg marker profiles were analyzed on Treg cells isolated from established orthotopic GL261-MGH tumors: (**a**) treated with IgG2a control (n=7), αPD1(n=7), αCTLA4 (n=6), or αPD1+αCTLA4 (n=6) on day 10 as well as from (**b**) draining cervical lymph nodes, (**c**) spleens and (**d**) the blood from the same animals (n=5-7). Statistical assessment was performed using one-way ANOVA with the Tukey adjustment for multiple comparisons. *$p < 0.05$, **$p < 0.01$, ***$p < 0.001$. N represents biological replicates. Data presented are mean ± SEM. Source data are provided as a Source Data file.

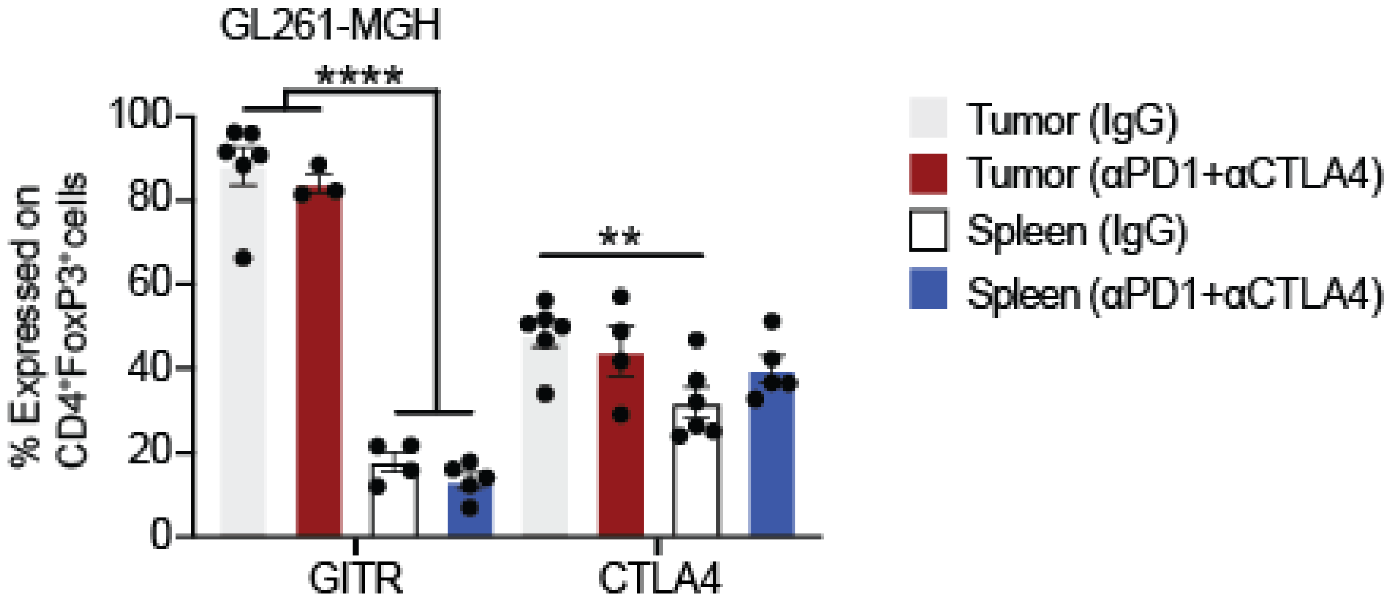

Supplementary Figure 7. GITR is predominantly expressed on GBM Treg cells.

A) Comparison of GITR and CTLA4 expression in GBM-infiltrating and splenic Treg cells in control IgG2a-treated (n=7) versus αPD1+αCTLA4-treated mice (n=6). Statistical assessment was performed using one-way ANOVA with the Tukey adjustment for multiple comparisons. Significance is shown as *$p < 0.05$, **$p < 0.01$, ***$p < 0.001$. N represents biological replicates. Data presented are mean ± SEM. Source data are provided as a Source Data file.

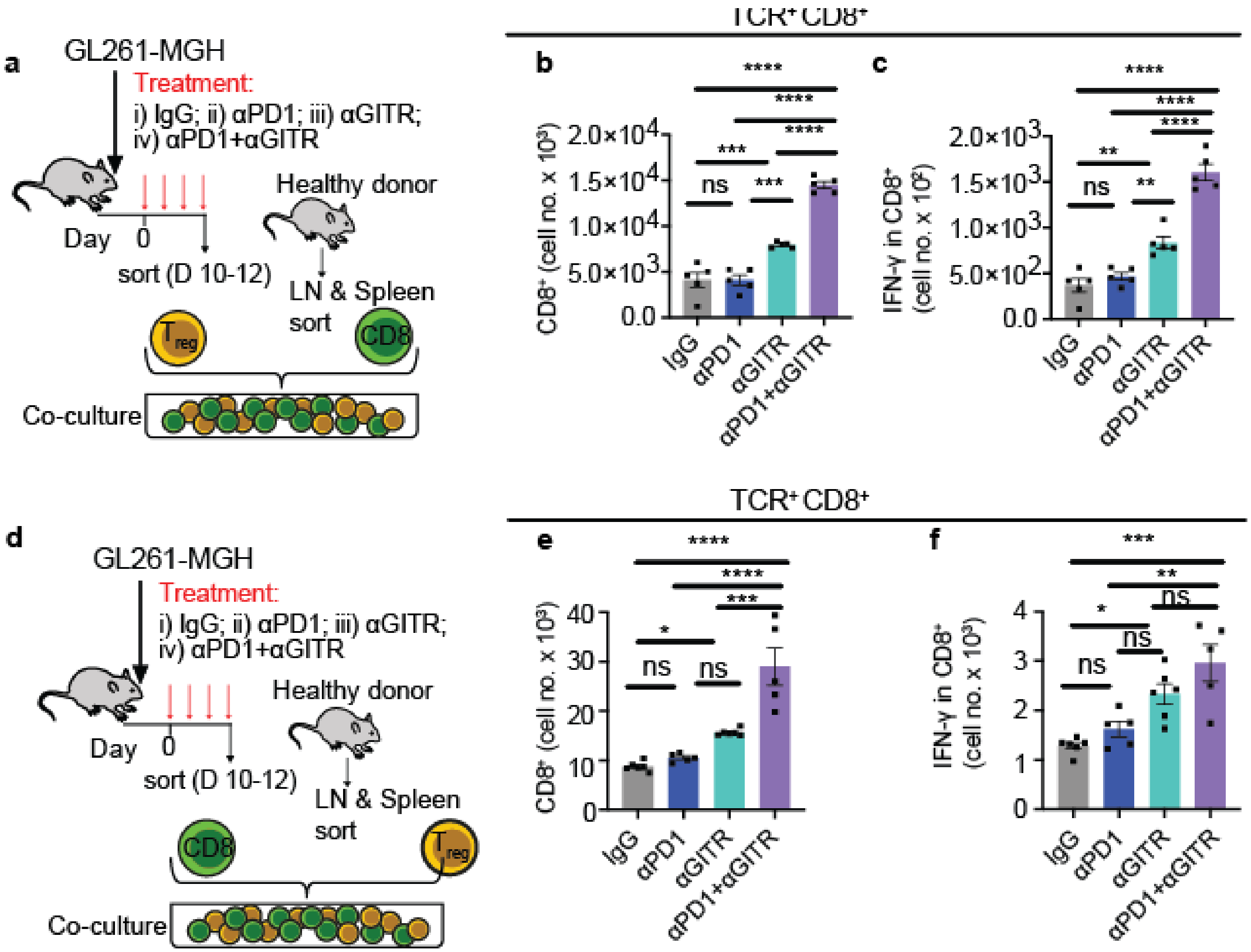

Supplementary Figure 8. Engagement of GITR with an agonistic antibody (αGITR) reprograms Treg cells to CD4 T effector cells.

a) Schematic representation of the ex vivo treatment experiments of Treg cells derived from tumor-bearing mice and CD8 T cells derived from non-tumor-bearing mice. Tumors were inoculated and randomized to treatment groups when tumor size reached ~2 mm^3. Treg cells were isolated from Spleen and cervical lymph nodes (CLN) of GL261-MGH bearing mice that had been treated with 4 doses of IgG2a (isotype control), ii) αPD1, iii) αGITR, and iv) αGITR + αPD1 (dose: 250 µg per antibody, day 10; 7-12 mice per group). CD8 T cells were harvested from healthy donor mice. Treg cells were enriched ($5x10^4$) and co-cultured with activated CD8 T cells (CD3/CD28 activation beads, $5x10^4$) (n=6) in 200uL volume. Data shown are representative of two independent repeats of experiments. N represents biological replicates.

b-c) CD8 T cells were analyzed after 3-4 days of co-culture for their number (ability to proliferate) **(b)** (IgG2a control (n=5), αPD1 (n=5), αGITR (n=5) or αPD1+αGITR (n=5)) p<0.0001 by one-way ANOVA test, and corrected for multiple comparisons using the Tukey adjustment. *p < 0.05, **p < 0.01, ***p < 0.001, ***p < 0.0001; and fraction of IFNγ expressing cells (**c**) (IgG2a control (n=6), αPD1 (n=5), αGITR (n=6) or αPD1+αGITR (n=5), p<0.0001 by one-way ANOVA test, and corrected for multiple comparisons using the Tukey adjustment. *p < 0.05, **p < 0.01, ***p < 0.001, ****p < 0.0001. Data shown are mean ± SEM, and are representative of two independent repeats of experiments. N represents biological replicates.

d) Schematic representation of the ex vivo experiments with heathy donor Treg cells and CD8 T cells derived from tumor-bearing mice treated with the indicated antibodies in vivo. CD8 T cells were harvested from the spleen and CLN of GL261-MGH bearing mice that had been treated with 4 doses of IgG2a (isotype control), ii)

αPD1, iii) αGITR, and iv) αGITR + αPD1 (dose: 250 µg, day 10; 6 mice per group). These CD8 T cells were co-cultured with Treg cells isolated from the spleen of healthy donor mice (n=10). N represents biological replicates. **e-f**) CD8 cells ((IgG n=6, αPD1 n=5, αGITR n=6, αPD1+ αGITR n=5) were analyzed after 3-4 days of co-culture for their ability to proliferate (**e**) and produce IFNγ (**f**). $p<0.0001$ (**e**) and $p=0.0002$ (**f**) by one-way ANOVA test, and corrected for multiple comparisons using the Tukey adjustment. Significance is shown as *$p < 0.05$, **$p < 0.01$, ***$p < 0.001$, ****$p < 0.0001$. Data presented are mean ± SEM. N represent biological replicates. Source data are provided as a Source Data file.

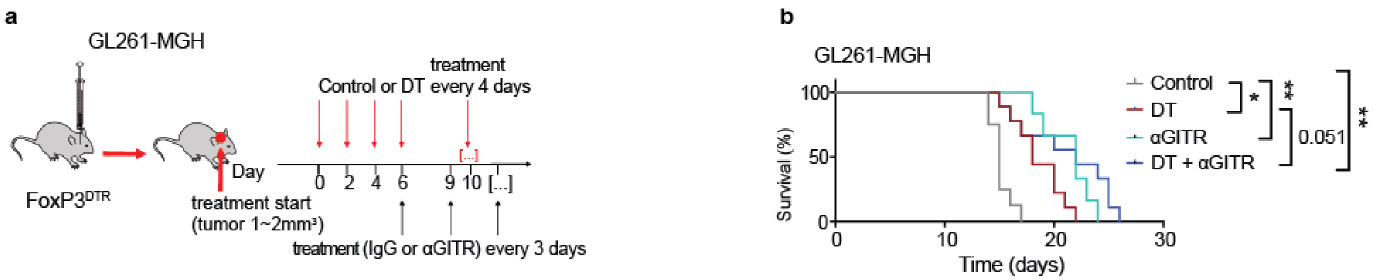

Supplementary Figure 9. Targeting GITR enhances survival benefit in GBM-bearing mice even when the number of Treg cells is reduced upon partial Treg cell depletion.

a) Schematic representation of experimental protocol to evaluate potential therapeutic effects of combining partial Treg cell depletion and Treg cell reprogramming using *FoxP3^{DTR}* mice and αGITR antibody, respectively. *FoxP3^{DTR}* mice bearing GL261-MGH tumors were treated with i) PBS control + IgG2a (n=8), ii) DT+IgG2a (n=9), iii) αGITR (n=6) or iv) DT and αGITR (n=9). DT therapy initiated at the time of randomization and was given every other day four times, followed by a maintenance dose every 4 days afterwards. αGITR therapy was initiated after the first 4 doses of DT, every 3 days afterwards. Data shown are representative of two independent repeats of experiments. N represents biological replicates.
b) survival of mice treated as in (**a**). Kaplan-Meier survival estimates in panel **b** were compared using the Mantel-Cox log-rank test as well as the Gehan-Breslow-Wilcoxon test. Significance is shown as $^{*}p < 0.05$, $^{**}p < 0.01$. Source data are provided as a Source Data file.

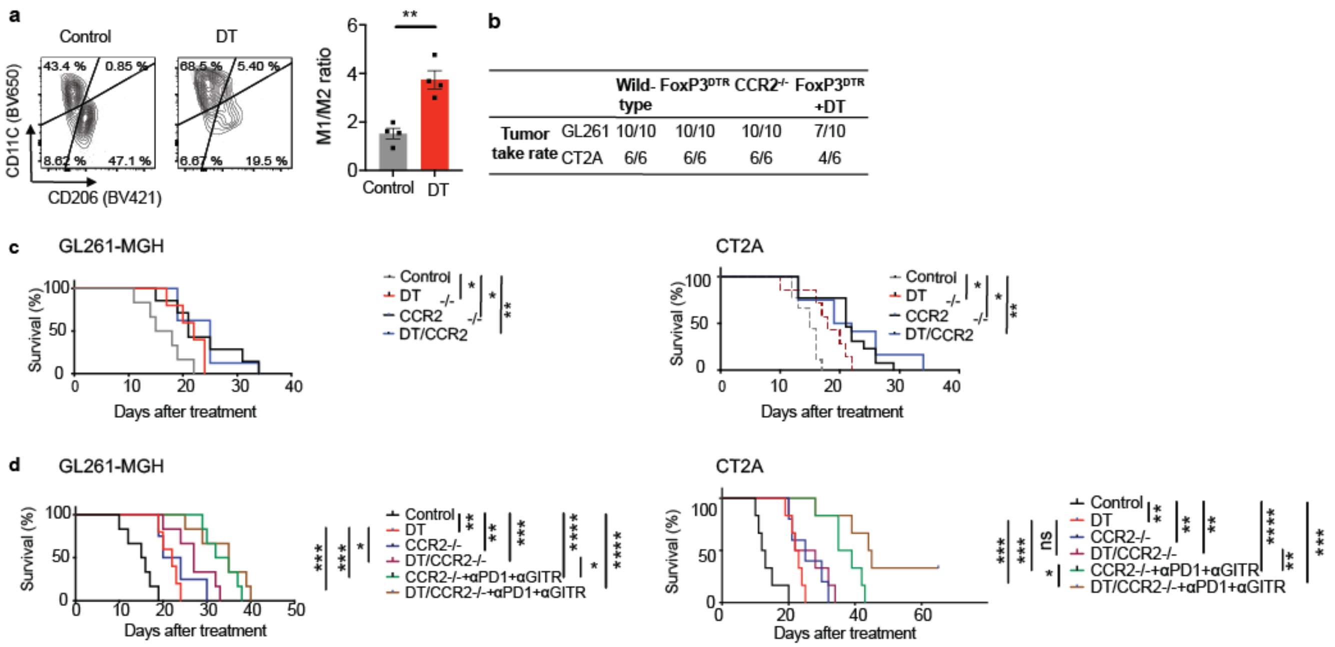

Supplementary Figure 10. Modulating macrophage recruitment and/or reprogramming does not cooperate with Treg cell reduction in improving survival outcome in mice bearing GBM tumors.

a) Gating strategy (left) and the ratio of M1-like to M2-like tumor-associated macrophages (TAMs) after Treg cell depletion, as indicated in the treatment protocol (for Supplementary Figure S4. Data shown are combined from two independent repeats of experiments. Data was analyzed using unpaired Two-tailed t-tests., p=0.0021. N represents biological replicates.

b) Tumor take rate of GL261-MGH and CT2A GBMs in CCR2$^{-/-}$ mice, mice with systemic Treg cell depletion (DT+*FoxP3*^{DTR} pre-tumor inoculation). Data shown are combined from two independent repeats of experiments. N represents biological replicates.

c) Survival of GL261-MGH and CT2A bearing mice (*FoxP3*^{DTR}, *CCR2*$^{-/-}$, and F*oxP3*^{DTR}/*CCR2*$^{-/-}$) treated with protocol shown in Fig. 4a. GL261-MGH [*CCR2*$^{-/-}$ (n=13), *FoxP3*^{DTR} (n=9), *FoxP3*^{DTR} treated with DT (n=7), F*oxP3*^{DTR}/*CCR2*$^{-/}$ treated with DT (n=12)]; and CT2A [*CCR2*$^{-/-}$ (n=7), *FoxP3*^{DTR} (n=6), *FoxP3*^{DTR} treated with DT (n=5), F*oxP3*^{DTR}/*CCR2*$^{-/}$ treated with DT (n=8)]; Kaplan-Meier survival estimates were compared using the Mantel-Cox log-rank test as well as the Gehan-Breslow-Wilcoxon test. Significance shown as as *p < 0.05, **p < 0.01. Data are representative of 2-4 independent analyses**.** Data shown are combined from two independent repeats of experiments. N represents biological replicates.

d) Survival of GL261-MGH and CT2A bearing mice (*FoxP3*^{DTR}, *CCR2*$^{-/-}$, and *FoxP3*^{DTR}/*CCR2*$^{-/-}$) with addition of αPD1+αGITR therapy. GL261-MGH [Control (n=6), DT (n=5), *CCR2*$^{-/-}$ (n=4), DT/*CCR2*$^{-/-}$(n=6), *CCR2*$^{-/-}$ +αPD1+αGITR(n=6), and DT/*CCR2*$^{-/-}$+αPD1+αGITR (n=6)], CT2A [Control (n=6), DT (n=6), *CCR2*$^{-/-}$ (n=5), DT/*CCR2*$^{-/-}$(n=6), *CCR2*$^{-/-}$+αPD1+αGITR(n=6), and DT/*CCR2*$^{-/-}$+αPD1+αGITR (n=6)]. Kaplan-Meier survival estimates were compared using the Mantel-Cox log-rank test as well as the Gehan-Breslow-Wilcoxon test. Significance shown as *p < 0.05, **p < 0.01, ***p < 0.001, ****p < 0.0001. Data is representative of 2 independent analyses**.** N represents biological replicates.

Source data are provided as a Source Data file.

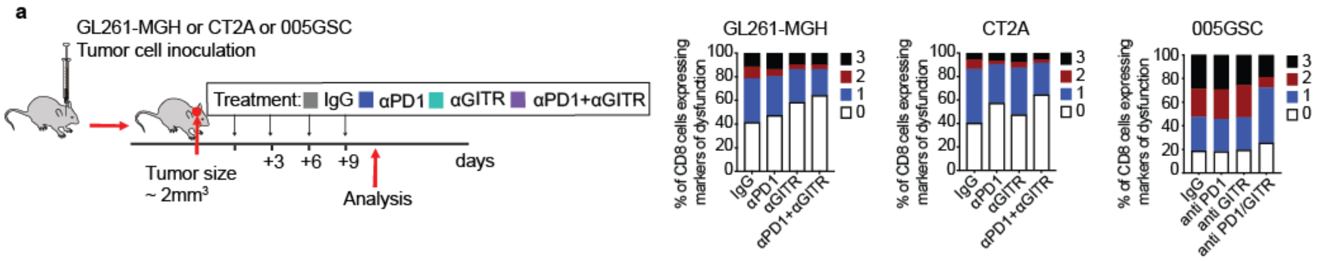

Supplementary Figure 11. αGITR + αPD1 therapy reduces CD8 T cell dysfunction.

Schematic representation of experimental protocol. Mice bearing orthotopic GBM tumors (GL261-MGH, CT2A or 005GSC, size ~ $2mm^3$) were treated with 4 doses of i) IgG2a, ii) αPD1, iii) αGITR, and iv) αGITR + αPD1 (250 µg/mice) and their intratumoral T cells were analyzed for expression of multiple CD8 T cell inhibitory markers [0: no inhibitory marker; 1: one marker of exhaustion (PD1, Lag3, or TIGIT); 2: (PD1/Lag3, PD1/TIGIT, or TIGIT/Lag3); 3: (PD1/Lag3/TIGIT)]. GL261-MGH [IgG2a control (n=4), αPD1 (n=5), αGITR (n=4) or αPD1+αGITR (n=7)], CT2A [IgG2a control (n=4), αPD1 (n=5), αGITR (n=6) or αPD1+αGITR (n=5)], and 005GSC CT2A [IgG2a control (n=6), αPD1 (n=6), αGITR (n=6) or αPD1+αGITR (n=6)].

Data shown are combined from two independent repeats of experiments. N represents biological replicates. Source data are provided as a Source Data file.

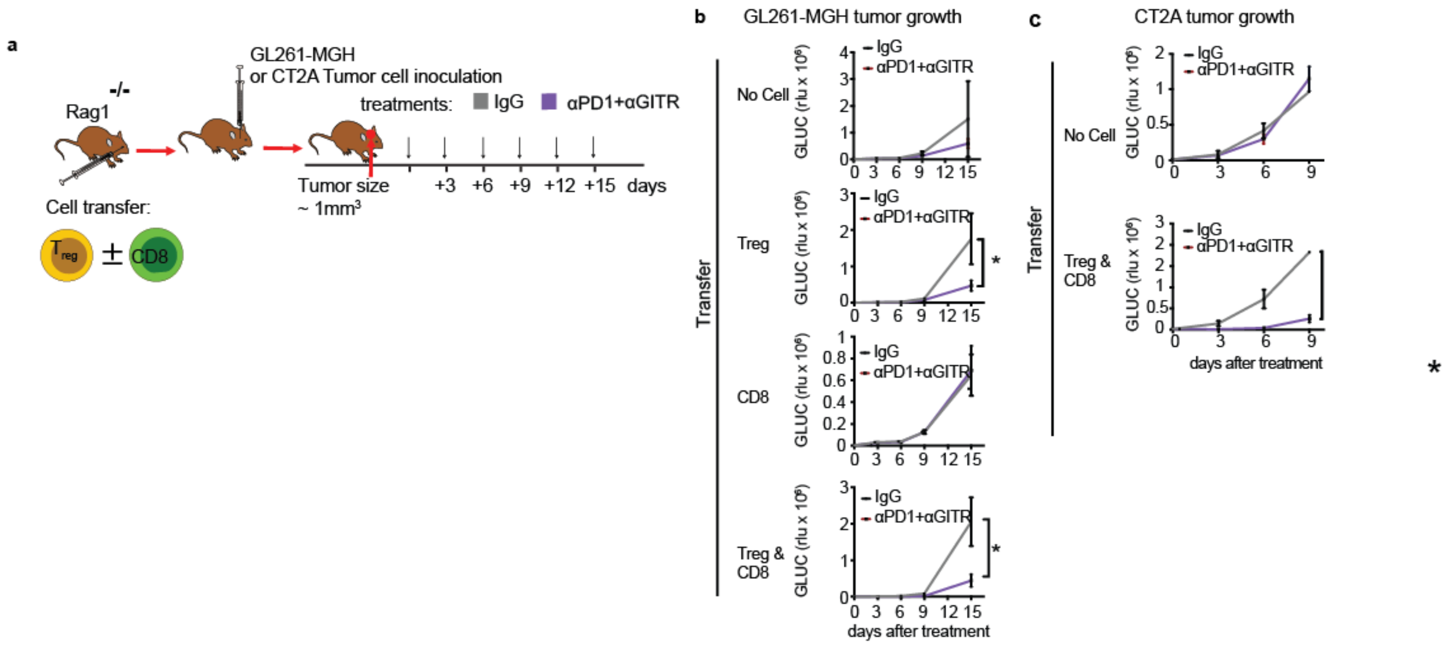

Supplementary Figure 12. CD4 Treg cells are sufficient to elicit the therapeutic effect of αPD1+αGITR.

a) Schematic representation of the experimental protocol to evaluate the contributions of CD8 and Treg cells to antitumor activity against GBM during αPD1+αGITR treatment. N represent biological replicates.

b-c) Purified and sorted CD8 T cells ($1x10^6$), Treg cells ($5x10^5$), or both were transferred to *Rag1*$^{-/-}$ mice 2 days before intracranial inoculation with tumor cells [(**b**) GL261-MGH or (**c**) CT2A]. Mice were treated [(GL261-MGH no cell: IgG n=5, αPD1+αGITR n=5)], [(GL261-MGH CD8 T cells: IgG n=5, αPD1+αGITR , n=5)] , [(GL261-MGH Treg cells: IgG n=5; αPD1+αGITR , n=6)], [(GL261-MGH CD8+Treg cells: IgG n=5; αPD1+αGITR , n=5)]; [(CT2A no cell: n=5, IgG; αPD1+αGITR , n=4)] , [(CT2A CD8+Treg cells: IgG n=4; αPD1+αGITR , n=4)]. with 6 doses of IgG2a (250 µg/mouse) or αPD1+αGITR (250 µg/mouse) and tumor growth was measured. P-values for tumor growth curves were calculated using unpaired Two-tailed t-tests for the last measured time points in the experiment. *p < 0.05, **p < 0.01, ***p < 0.001 and lower. P=0.08 for GL261-MGH bearing mice transferred with Treg cells, P=0.047 for GL261-MGH bearing mice transferred with Treg cells+CD8 T cells, and P=0.012 for CT2A bearing mice transferred with Treg cells+CD8 T cells. Other p values were above 0.05 and thus, statistically insignificant. Data presented are mean ± SEM. N represents biological replicates. Source data are provided as a Source Data file.

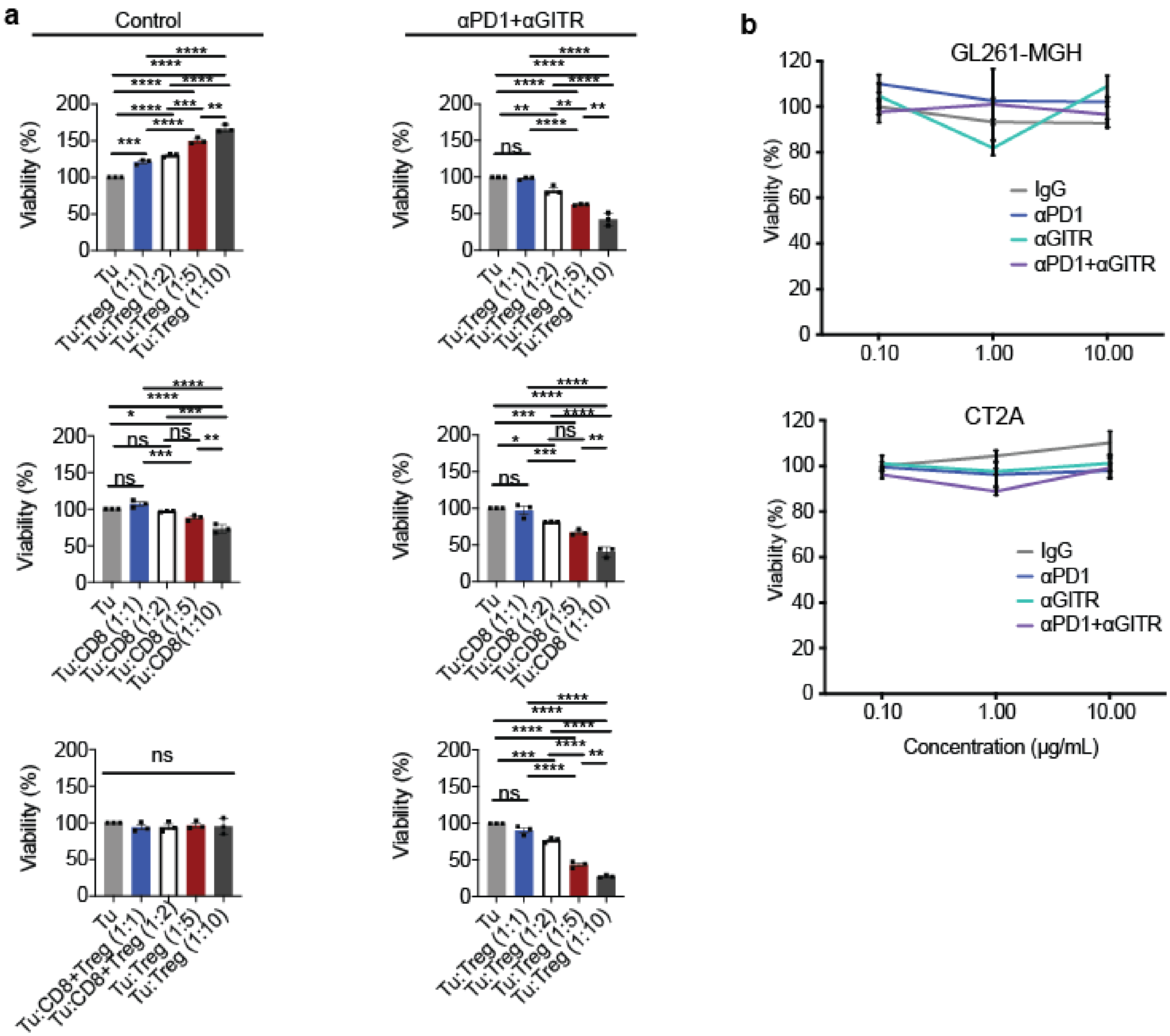

Supplementary Figure 13. Converted Treg cells cooperate with CD8 T cells in killing tumor cells.

a) GL261-MGH tumor cell viability after co-culture with Treg cells and CD8 T cells and treatment with IgG2a or αPD1+αGITR. Treg cells and CD8 T cells were sorted from spleens of naive mice (n=4 per condition) and co-cultured with GL261-MGH tumor cells for 48 hours at indicated ratios. Data shown are mean ± SEM. Each condition is tested as 3 biological replicates. All p-values were calculated using one-way ANOVA test [control (Treg cells $p<0.0001$, CD8 $p<0.0001$, and Treg cells+CD8 $p<0.8358$], [αPD1+αGITR (Treg cells $p<0.0001$), CD8 $p<0.0001$, Treg cells +CD8 $p<0.0001$)] and were corrected for multiple comparisons using the Tukey adjustment. Significance shown as *$p < 0.05$, **$p < 0.01$, ***$p < 0.001$, ***$p < 0.0001$.

b) Evaluation of effects of antibody treatment alone on GBM tumor cell viability. GL261-MGH or CT2A cells were seeded in a 96 well plate (20,000 cells/60 µl/well) in quadruplicates. Cells were treated with 0.1, 1 and 10 µg/ml of αGITR, αPD1, αPD1+αGITR or control (IgG2a). Cell viability was measured after 72 h with the MTT assay. Each condition is tested as 3 biological replicates. Data presented are mean ± SEM. All p-values were calculated using one-way ANOVA test, and found to be insignificant.

Source data are provided as a Source Data file.

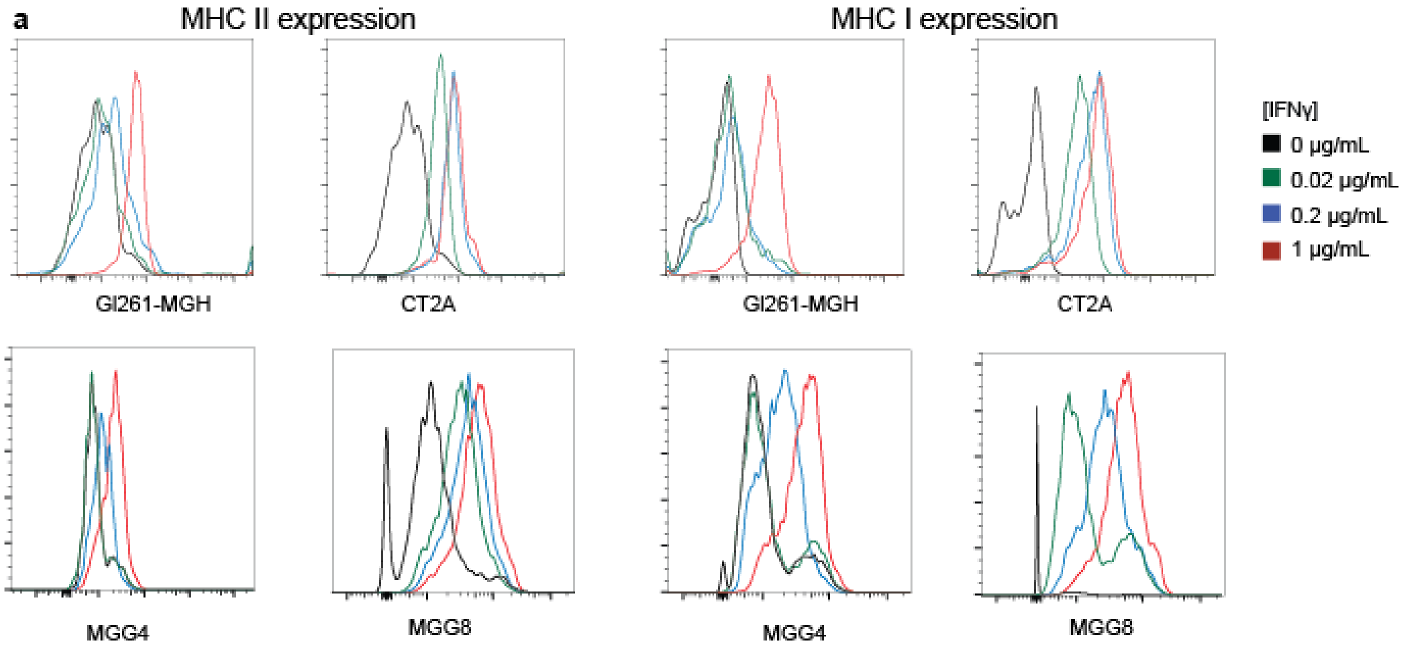

Supplementary Figure 14. Exposure to IFNγ induces MHC Class I and II expression on mouse and human GBM cells

Flow cytometric measurement of expression of MHC class I and MHC class II on GL261-MGH (mouse), CT2A (mouse), MGG4 (human), and MGG8 (human) GBM cells after treatment with IFNγ *in vitro*. Each condition is tested as 3 biological replicates. Source data are provided as a Source Data file.

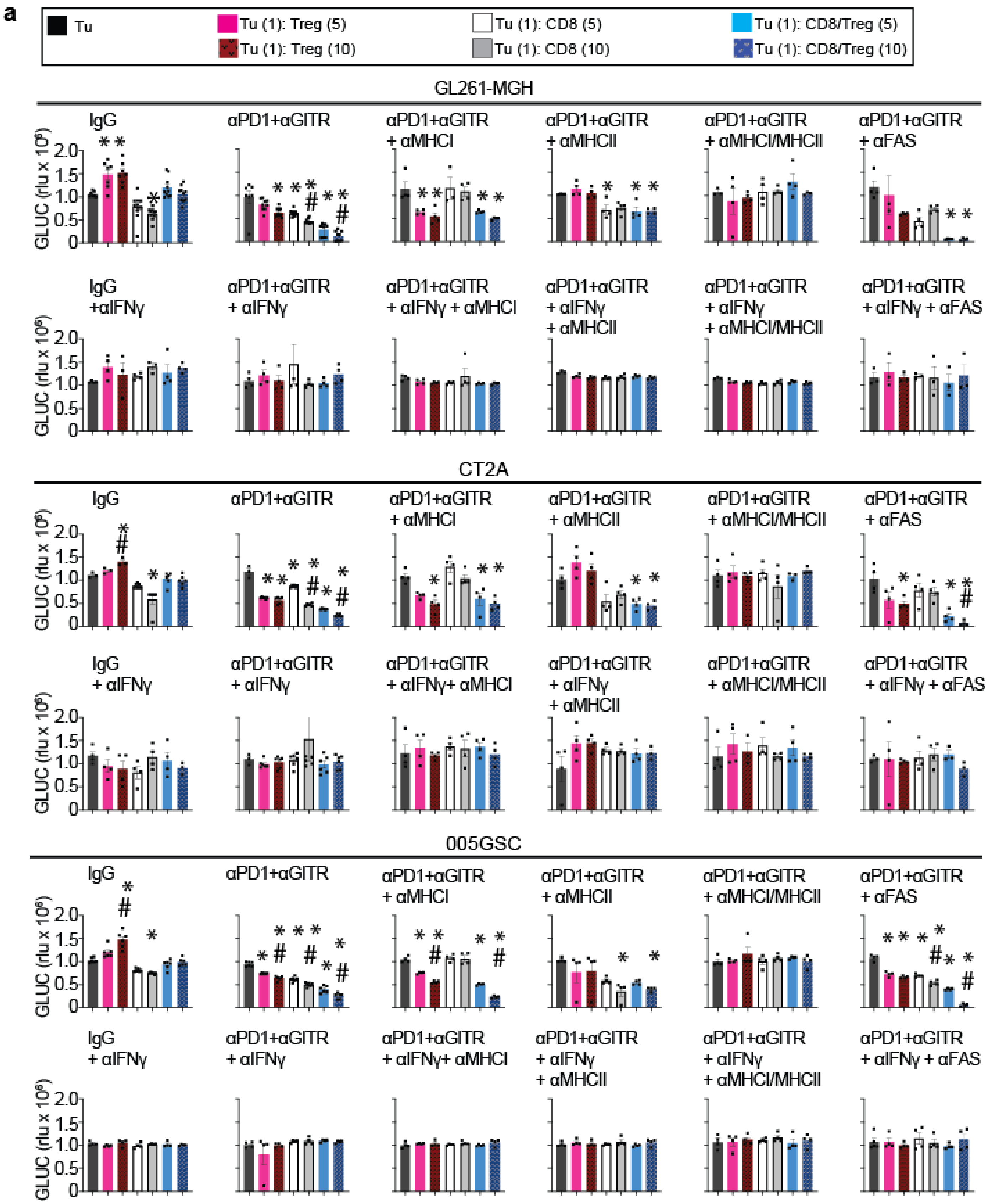

Supplementary Figure 15. Converted Treg cells cooperate with CD8 T cell function in killing tumor cells in an IFNγ- and MHC-dependent manner.

Tumor cells (GL261-MGH, CT2A or 005GSC, 1x10^3 cells) were co-cultured for 48 hours with Treg cells, CD8 T cells or both (as described above) with 1:5 or 1:10 ratio. GLUC activity in the cell culture media was measured

and used as a surrogate marker for tumor cell proliferation. Tumor cell viability was measured under treatment with i) IgG, ii) αPD1+αGITR, ii) αPD1+αGITR+αMHCI, iii) αPD1+αGITR+αMHCII, iv) αPD1+αGITR +αMHCI&II, v) αPD1+αGITR+αFAS and each therapy combined with αIFNγ. Data presented are mean ± SEM. All p-values were calculated using one-way ANOVA test and corrected for multiple comparisons using the Tukey adjustment. one-way ANOVA test for GL261-MGH [IgG (p<0.0001), αPD1+αGITR (p<0.0001), αPD1+αGITR+αMHCI (p=0.0001), αPD1+αGITR+αMHCII (p<0.0001), αPD1+αGITR +αMHCI&II (p=0.5522), αPD1+αGITR+αFAS (p=0.0006), IgG+αIFNγ (p=0.4244), αPD1+αGITR+αIFNγ (p=0.6307), αPD1+αGITR+αMHCI+αIFNγ (p=0.4374), αPD1+αGITR+αMHCII+αIFNγ (p=0.0039), αPD1+αGITR+αMHCI&II+αIFNγ (p=0.0035), αPD1+αGITR+αFAS+ αIFNγ (p=0.9803); one-way ANOVA test for CT2A [IgG (p<0.0001), αPD1+αGITR (p<0.0001), αPD1+αGITR+αMHCI (p<0.0001), αPD1+αGITR+αMHCII (p<0.0001), αPD1+αGITR +αMHCI&II (p=0.6248), αPD1+αGITR+αFAS (p<0.0001), IgG+αIFNγ (p=0.4427), αPD1+αGITR+αIFNγ (p=0.4542), αPD1+αGITR+αMHCI+αIFNγ (p=0.8718), αPD1+αGITR+αMHCII+αIFNγ (p=0.1502), αPD1+αGITR+αMHCI&II+αIFNγ (p=0.7485), αPD1+αGITR+αFAS+ αIFNγ (p=0.9340); one-way ANOVA test for 005GSC [IgG (p<0.0001), αPD1+αGITR (p<0.0001), αPD1+αGITR+αMHCI(p=0.0137), αPD1+αGITR+αMHCII (p<0.0001), αPD1+αGITR +αMHCI&II (p=0.6324), αPD1+αGITR+αFAS (p<0.0001), IgG+αIFNγ (p=0.5353), αPD1+αGITR+αIFNγ (p=0.2929), αPD1+αGITR+αMHCI+αIFNγ (p=0.5353), αPD1+αGITR+αMHCII+αIFNγ (p=0.4649), αPD1+αGITR+αMHCI&II+αIFNγ (p=0.9158), αPD1+αGITR+αFAS+ αIFNγ (p=0.7624). *$p < 0.05$. * represents statistical difference from control (Tu). # marks significance differences between similar groups with different ratio of T cell to tumor cells (Tu) e.g., [Tu (1) : CD8 (5)] versus [Tu (1) : CD8(10)]. Each condition was tested as at least 3 biological replicates. Source data are provided as a Source Data file.

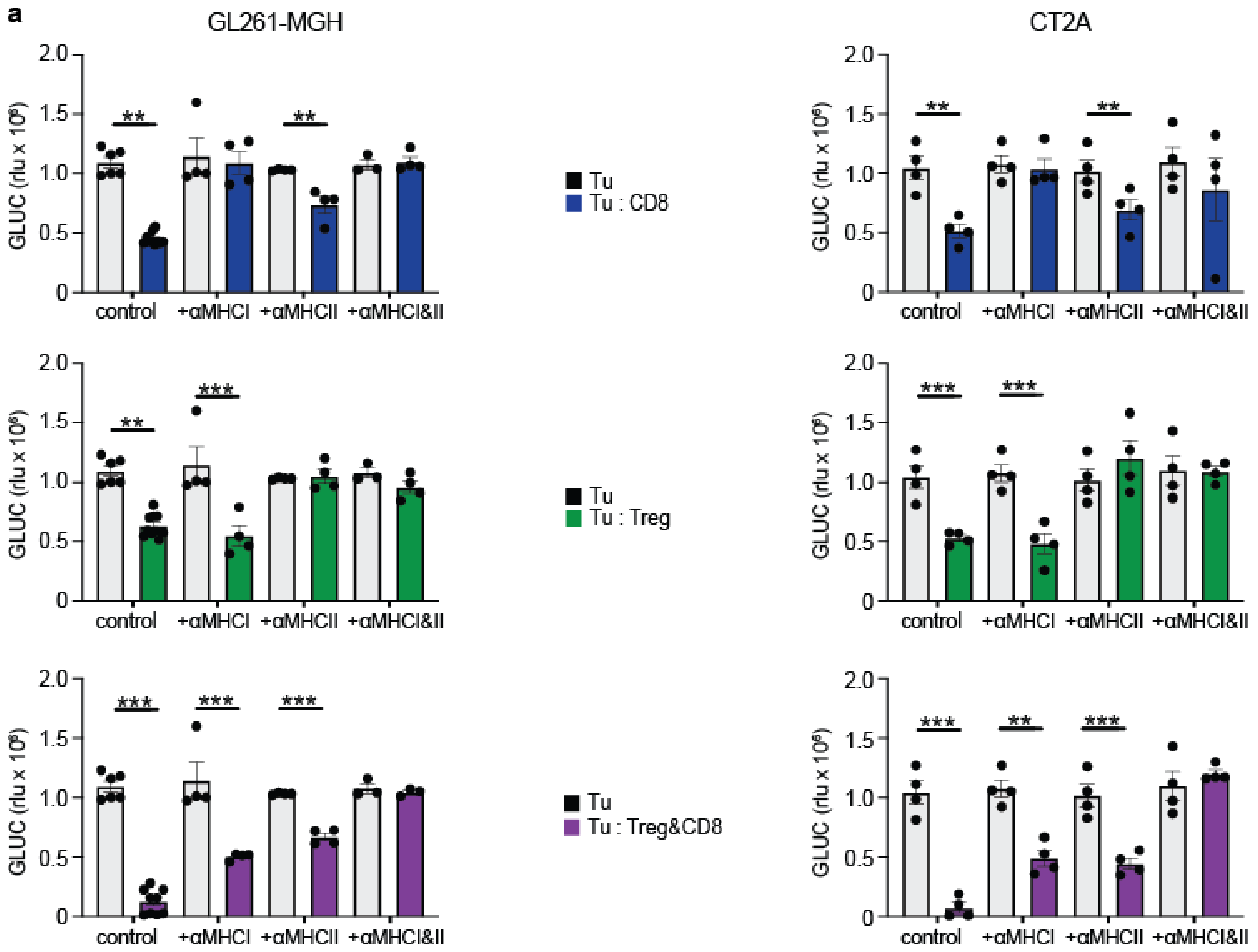

Supplementary Figure 16. MHC dependent tumor cell recognition is required for the anti-tumor activity of reprogrammed Treg cells and reactivated CD8 T cells by αPD1+αGITR therapy

a) Treg cells and CD8 T cells were isolated from spleens of healthy donor mice (n=6 per condition). Tumor cells ($1X10^3$ cells) were co-cultured for 48 hours with Treg cells, CD8 T cells, or Treg cells and CD8 T cells in ratios of 1:10. GLUC activity in the cell culture media was measured and used as a surrogate marker for tumor cell viability. Tumor cell viability was measured under treatment with IgG2a or αPD1+αGITR with and without blockade of MHC Class I, MHC Class II, combined MHC Class I & II for Treg cells, CD8 T cell or Treg cells+CD8 T cells. Data is representative of 2 independent experiments. Data presented are mean ± SEM. All p-values were calculated using a two-sided t-test. *$p < 0.05$, **$p < 0.01$, ***$p < 0.001$. Each condition was tested as 3 biological replicates. Source data are provided as a Source Data file.

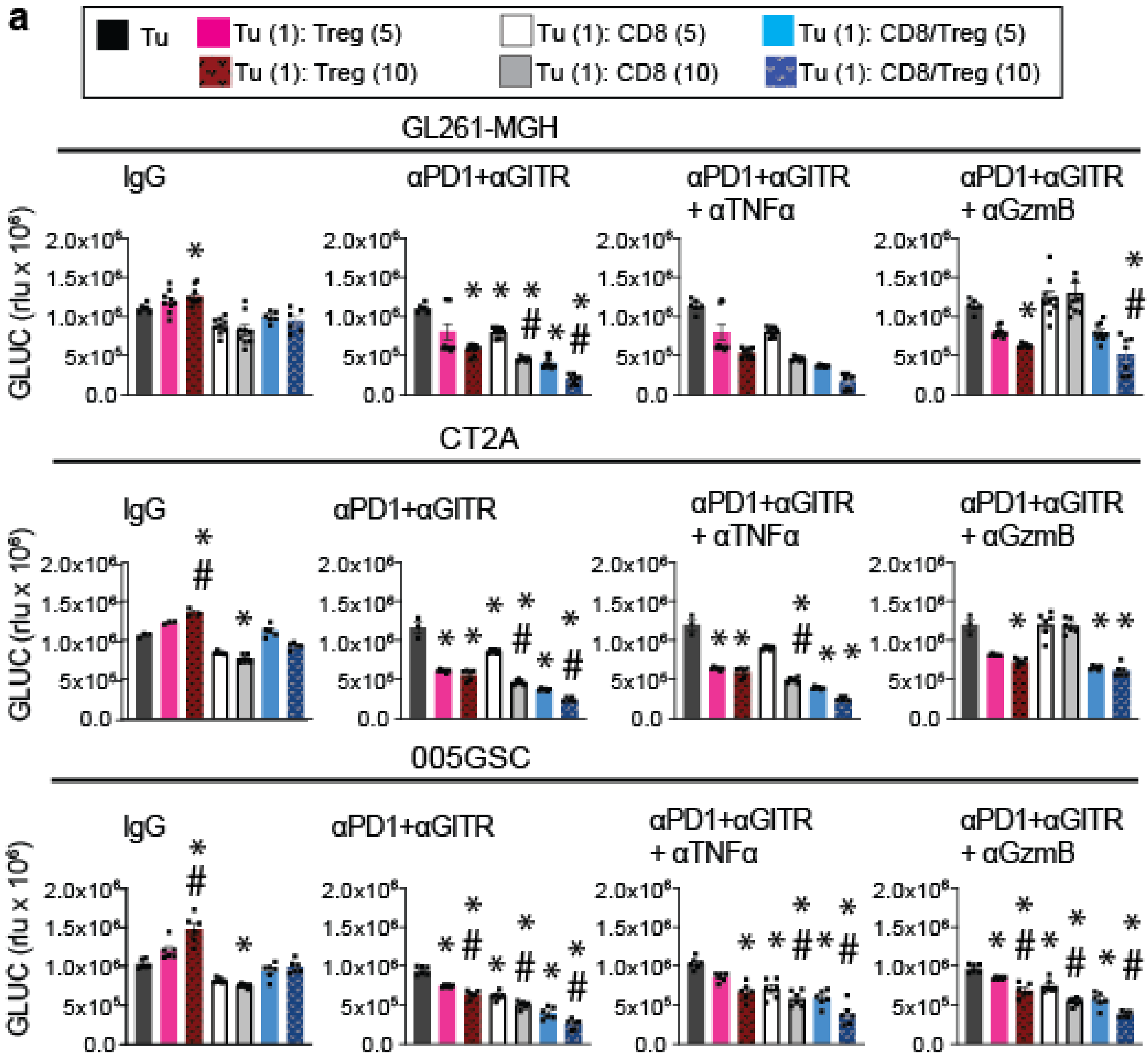

Supplementary Figure 17. Anti-tumor function of reprogrammed Treg cells and CD8 T cells remained unchanged under TNFα blockade, while GzmB blockade reduced the CD8 T cell anti-tumor activity without affecting the reprogrammed Treg cells.

Tumor cells (GL261-MGH, CT2A or 005GSC, $1x10^3$ cells) were co-cultured for 48 hours with Treg cells or CD8 T cells or both (as described in Supplementary Figure 15) with 1:5 or 1:10 ratio. GLUC activity in the cell culture media was measured and used as a surrogate marker for tumor cell proliferation. Tumor cell viability was measured under treatment with i) IgG2a, ii) αPD1+αGITR, iii) αPD1+αGITR+αTNFα, and iv) αPD1+αGITR+αGzmB. Data presented are mean ± SEM. All p-values were calculated using one-way ANOVA test and corrected for multiple comparisons using the Tukey adjustment. *p < 0.05. * represents statistical difference from control (Tu). One-way ANOVA test for GL261-MGH [IgG (p<0.0001), αPD1+αGITR (p<0.0001), αPD1+αGITR+αTNFα (p<0.0001), αPD1+αGITR+αGzmB (p<0.0001)], one-way ANOVA test for CT2A [IgG (p<0.0001), αPD1+αGITR (p<0.0001), αPD1+αGITR+αTNFα (p<0.0001), αPD1+αGITR+αGzmB

(p<0.0001)], and one-way ANOVA test for 005GSC [IgG (p<0.0001), αPD1+αGITR (p<0.0001), αPD1+αGITR+αTNFα (p<0.0001), αPD1+αGITR+αGzmB (p<0.0001)]. # marks significant differences between similar groups with different ratio of T cell to tumor cells (Tu) e.g., [Tu (1) : CD8 (5)] versus [Tu (1) : CD8(10)]. Each condition is tested as 3 biological replicates. Source data are provided as a Source Data file.

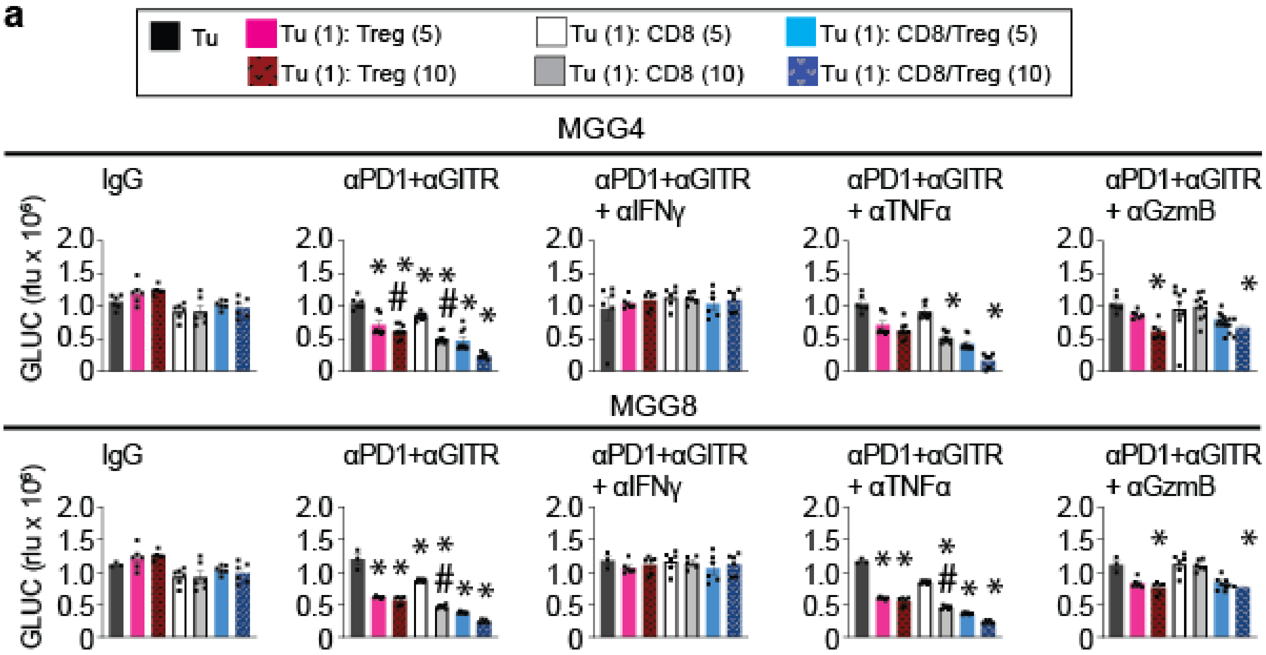

Supplementary Figure 18. αPD1+αGITR therapy enabled anti-tumor function of Treg cells and CD8 T cells depends on IFNγ and not on GzmB or TNFα.

Tumor cells (MGG4 or MGG8, 1x10^3 cells) were co-cultured for 72 hours with human Treg cells or CD8 T cells or both harvested from peripheral blood, with 1:5 or 1:10 ratio. GLUC activity in the cell culture media was measured and used as a surrogate marker for tumor cell proliferation. Tumor cell viability was measured under treatment with i) IgG2a, ii) αPD1+αGITR, iii) αPD1+αGITR+αIFNγ iv) αPD1+αGITR+αTNFα, and v) αPD1+αGITR+αGzmB. Data presented are mean ± SEM. All p-values were calculated using one-way ANOVA test and corrected for multiple comparisons using the Tukey adjustment. One-way ANOVA test for MGG4 [IgG ($p=0.0007$), αPD1+αGITR ($p<0.0001$), αPD1+αGITR+αIFNγ ($p=0.8207$), αPD1+αGITR+αTNFα ($p<0.0001$), αPD1+αGITR+αGzmB ($p=0.0014$)], one-way ANOVA test for MGG8 [IgG ($p=0.0008$), αPD1+αGITR ($p<0.0001$), αPD1+αGITR+αIFNγ (0.8121), αPD1+αGITR+αTNFα ($p<0.0001$), αPD1+αGITR+αGzmB ($p<0.0001$)],]
*$p < 0.05$. # marks statistically significant differences between similar groups with different ratio of T cell to tumor cells (Tu) e.g., [Tu (1): CD8 (5)] versus [Tu (1) : CD8 (10)]. Each condition is tested as 3 biological replicates. Source data are provided as a Source Data file.

<u>Supplementary References</u>

1. Stupp, R. *et al.* Radiotherapy plus concomitant and adjuvant temozolomide for glioblastoma. *The New England journal of medicine* **352**, 987-996 (2005).
